# Thyroid antibody positivity in pregnancy is associated with altered reproductive hormone concentrations and placental growth in Lewis rats

**DOI:** 10.1113/JP291573

**Published:** 2026-07-25

**Authors:** Rebecca L. Brady, Nykola L. Kent, Anna G. Reid, Liana P. Paszkowski, Ashley S. Meakin, Dayna A. Zimmerman, Dinithi T. Mahaliyanage, Elliott S. Neal, Michael D. Wiese, Helle Bielefeldt‐Ohmann, Janna L. Morrison, James S. M. Cuffe

**Affiliations:** ^1^ School of Biomedical Sciences The University of Queensland Brisbane Queensland Australia; ^2^ Early Origins of Adult Health Research Group, School of Pharmacy and Biomedical Science, Robinson Research Institute, College of Health Adelaide University Adelaide South Australia Australia; ^3^ Centre for Pharmaceutical Innovation, School of Pharmacy and Biomedical Science, College of Health Adelaide University Adelaide South Australia Australia; ^4^ School of Chemistry and Molecular Biosciences University of Queensland Brisbane Queensland Australia

**Keywords:** estrus, fetal growth, GDM, placental inflammation, TgAb, TPOAb

## Abstract

**Abstract:**

Thyroid antibody positivity (TAb+) is common among reproductive‐aged women, and TAb+ in pregnancy is associated with complications including miscarriage and gestational diabetes. These associations persist in women with normal thyroid status, but there are minimal studies examining the underlying mechanisms. This study was designed to investigate the effects of TAb+ on maternal, placental and fetal health, with a focus on metabolism, using a Lewis rat model of TAb+ induced before pregnancy. Aspects of maternal fertility, cardiovascular adaptations and placental function were also assessed. Oestrous cycling was monitored prior to pregnancy, and an intraperitoneal glucose tolerance test was performed on embryonic day (E)16. Rats were killed at late pregnancy (E20), with maternal and fetal plasma and tissues collected for analysis of metabolic, endocrine and placental function. TAb+ rats did not have altered glucose tolerance during pregnancy but did have increased oestrous cycle length before pregnancy and altered concentrations of key reproductive hormones at E20, including rat placental lactogen. Placentas exposed to TAb+ were larger, with increased expression of glucose transporters and inflammatory markers. TAb+ dams had a reduced proportion of male fetuses per litter but no changes in litter size or fetal growth. This study demonstrates that although TAb+ was not associated with severe changes to the maternal parameters investigated, several aspects of placental biology were impacted. These findings support further investigation of TAb+ as a thyroid hormone‐independent contributor to pregnancy complications, with placental function emerging as a potential mechanistic target.

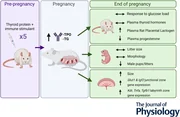

**Key points:**

- Thyroid antibody positivity (TAb+) is associated with an increased risk of complications during pregnancy, including miscarriage and gestational diabetes.
- Associations are often attributed to thyroid dysfunction, yet risks persist in euthyroid women. Research investigating the effects of TAb+ on glucose metabolism and placental function in models with normal thyroid hormone concentrations is required.
- In our rodent model of TAb+ during pregnancy, there were limited changes to glucose metabolism or litter size. However, we did see increased placental growth, altered rat placental lactogen and progesterone concentrations, and a reduced proportion of male fetuses.
- This study suggests the link between TAb+ and gestational diabetes is not driven by elevated antibodies. However, TAb+ impacts aspects of fertility, fetal survival and placental development, which has implications for other pregnancy disorders.
- The findings from this study highlight that thyroid antibodies are an important factor to consider when managing patients with TAb+ during pregnancy, outside of consequences to thyroid health.

## Graphical abstract

**Abstract figure legend** Thyroid antibody positivity (TAb+) in pregnancy is associated with adverse pregnancy outcomes, but the mechanisms are unclear. This study developed a rodent model of TAb+ in pregnancy, induced prior to mating so that TAb+ exposure occurred throughout the pregnancy. TAb+ did not alter thyroid hormone concentrations and had minimal effects on maternal glucose control and litter size. TAb+ rats had altered plasma rat placental lactogen and progesterone concentrations, and a lower proportion of male fetuses. Placentas exposed to TAb+ were larger, with increased gene expression of glucose transporters and inflammatory and vascular markers. Original figure created using Biorender (https://biorender.com).

## Introduction

Thyroid autoimmunity, characterised by the presence of autoantibodies against thyroid‐specific antigens, is the leading cause of hypothyroidism in iodine‐replete populations (Cooper & Biondi, 2012; Cuthrell et al., 2022). Thyroid antibodies (TAbs), most commonly against thyroglobulin (TgAb) and thyroid peroxidase (TPOAb), induce thyroid dysfunction by impairing the function of thyroid proteins and causing structural damage to the thyroid gland. This results in impaired thyroid hormone synthesis and compensatory increases in thyroid stimulating hormone (TSH) (Ragusa et al., 2019). High rates of TAb positivity (TAb+) are reported in reproductive‐aged and pregnant women, with one study reporting prevalence as high as 20% (Caturegli et al., 2014; Hu et al., 2022; McLeod & Cooper, 2012). A recent meta‐analysis of 25 cohort studies, including collective data from over 65,000 pregnant women, reported that TPOAb+ was associated with a 21‐fold increased prevalence of overt hypothyroidism and an eightfold increase in the rate of subclinical hypothyroidism (Osinga et al., 2024). Despite this, more than 80% of women with TPOAb+ showed no evidence of thyroid disease. Clinical studies have demonstrated that women with TAb+ have increased rates of infertility (Poppe et al., 2002), and TAb+ during pregnancy is linked to increased rates of miscarriage, pre‐term birth and gestational diabetes mellitus (GDM) (Chen et al., 2017; Karakosta et al., 2012; Thangaratinam et al., 2011). This increased risk of reproductive dysfunction remains even in women with normal thyroid hormone concentrations, which suggests that TAbs may play a more direct role outside of altered thyroid function (Abdolmohammadi‐Vahid et al., 2022).

There is a well‐established link between thyroid dysfunction and GDM, with pregnancies complicated by both subclinical and overt hypothyroidism showing increased rates of GDM (Luo et al., 2021). This suggests that both low thyroxine (T4) and high TSH are independent risk factors for GDM (Alifu et al., 2024). Given that TPOAb+ patients with subclinical hypothyroidism have an increased risk of progression to overt disease (Zhang et al., 2024), it is important to also investigate TAbs in the context of GDM. Recent systematic reviews have highlighted that an increased risk for GDM is only present in women with subclinical hypothyroidism if they also have TAb+ (Dincgez et al., 2024; Kent et al., 2021). This suggests that TAbs may be an important risk factor for dysregulated glucose homeostasis or insulin sensitivity during pregnancy, though limited research has focused specifically on the mechanisms linking TAb+ with GDM or other aspects of metabolic function. Given that the placenta is a key organ regulating maternal endocrine adaptations to pregnancy, one mechanism through which TAb+ may lead to GDM is through altered placental function.

Poor placental development and dysfunction are key factors in the pathogenesis of many pregnancy complications. Crosstalk between placental extravillious trophoblasts, vascular smooth muscle cells (VSMCs), decidual cells and immune cells facilitates spiral artery remodelling and implantation (James et al., 2011; Okada et al., 2018; Whitley & Cartwright, 2009). Deficits in these early processes are implicated in infertility and miscarriage as well as altered placental pathologies. Perturbed vascularisation and inflammation occurring later in pregnancy contribute to conditions such as fetal growth restriction and pre‐term birth (Scifres & Nelson, 2009; Suresh et al., 2022). However, the impact of TAbs on placental vascularisation and inflammation is poorly characterised. One study showed that TAb+ mice demonstrated reduced endometrial receptivity and implantation failure (Wu et al., 2019). Outside of pregnancy, primary cell‐culture studies highlight altered VSMC function after incubation with TPOAbs (Zhang et al., 2019). This may suggest similar mechanisms underlying the relationship between TAbs and both infertility and miscarriage, but consequences to fetal health cannot be fully determined without looking at later stages of pregnancy.

No studies have investigated the mechanisms by which TAbs impact maternal cardiometabolic health in pregnancy, or potential consequences to placental and fetal outcomes. This project aimed to explore the mechanisms by which TAbs alter placental function, and maternal and fetal metabolism, while looking at several markers of fertility. This is crucial for understanding how TAbs contribute to the development of pregnancy complications. Animal models of TAb+ are generated through immunisation with a thyroid protein, often in the presence of increased iodine exposure as a ‘second hit’ (Hou et al., 2018; Song et al., 2011). There are several existing models that have focused on early and mid‐gestation, so our study focused on outcomes in late pregnancy to determine the impact on fetal development. We hypothesised that pregnant rats with TAb+ would experience impaired glucose tolerance, smaller litter size and reduced placental efficiency linked with altered markers of vascularisation and inflammation, and impaired fetal growth.

## Methods

### Ethical approval

Ethical approval for this study was granted by the University of Queensland Anatomical Biosciences Animal Ethics Committee (2020/AE000438) and was conducted in accordance with the Australian Code of Practice for Care and Use of Animals for Scientific Purposes. All experiments were conducted in accordance with the principles set out by Grundy and O'Halloran (Grundy, 2015; O'Halloran, 2024).

### Experimental animal model

Twenty nulliparous 7–8‐week‐old female Lewis rats were purchased from the Australian Resource Centre (Murdoch, Western Australia). Sample sizes were decided based on power calculations prior to ethical approval. Rats were randomly assigned to either control (*n* = 10) or TAb+ (*n* = 10) groups and were housed in groups of 2–3 individually ventilated cages at the Australian Institute for Bioengineering and Nanotechnology animal facility at the University of Queensland. The facility's environment was controlled at 22 ± 2°C and 40 ± 5% humidity with an artificial 12 h light–dark cycle (lights on from 06.00 h to 18.00 h). Throughout the protocol, animals were monitored daily for welfare, food consumption and water consumption.

After at least 1 week of acclimatisation, on experimental day 1 (Ex1), all rats were given *ad libitum* access to a custom hand‐made diet made freshly twice a week; a diet we have previously published (Hofstee et al., 2019). This diet limits selenium to prevent oversupply of this nutrient while all other nutrients are optimised for rodent pregnancy studies. Rats in the control group were given *ad libitum* access to standard drinking water, and the TAb+ group were given *ad libitum* access to drinking water containing sodium iodide (NaI) (0.05% w/v, Sigma‐Aldrich, #217638). Selenium content was limited for both groups, but iodine was manipulated only in the TAb+ group, so that the effects of immunisation were exacerbated while offering a suitable control group.

**Figure 1:**
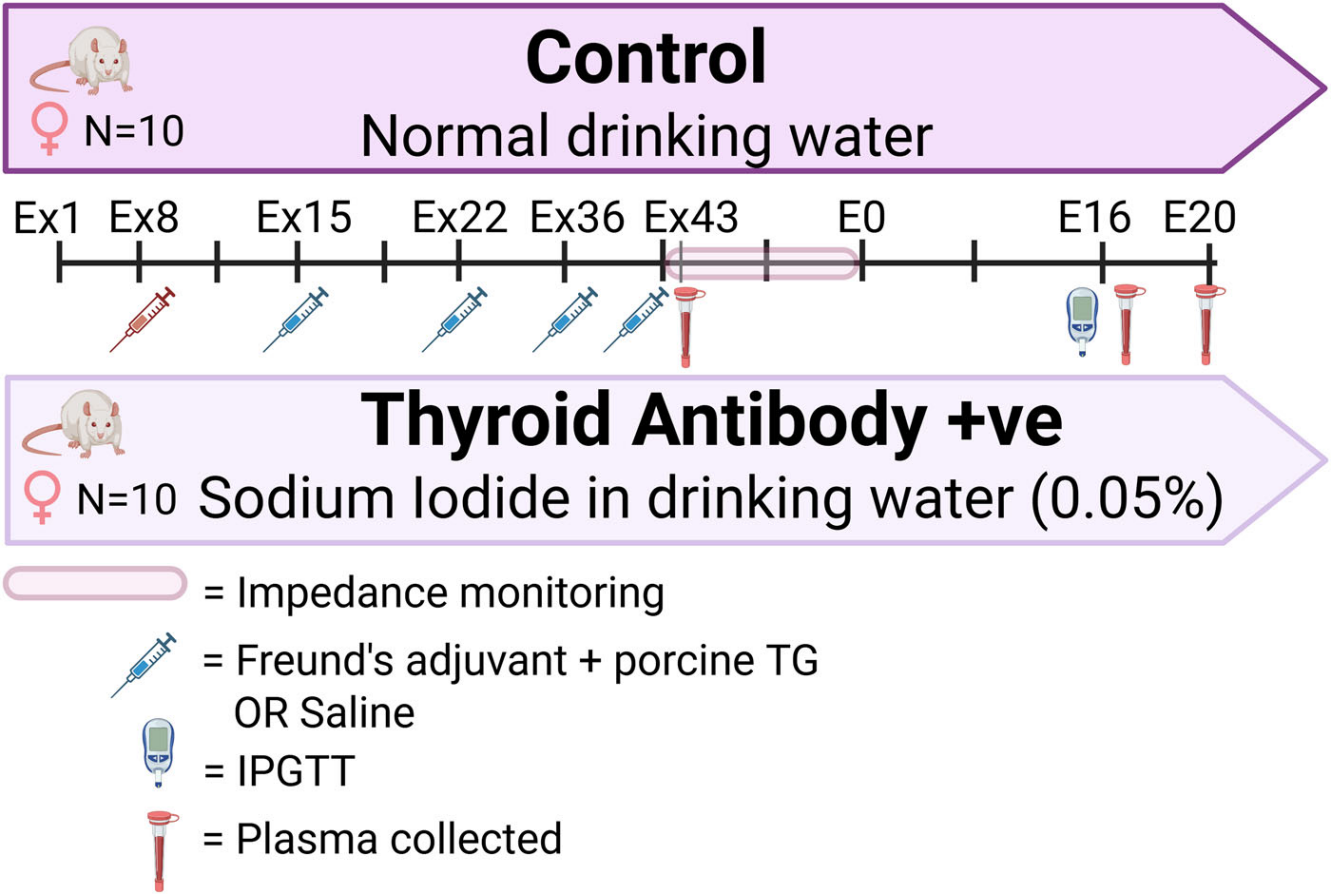
Rat model of thyroid antibody positivity (TAb+) during pregnancy Twenty 7–8week‐old Lewis rats were randomly assigned to control or treated (TAb+) groups. TAb+ rats had *ad libitum* access to sodium iodide in drinking water (0.05% w/v) for the duration of the protocol and were injected with porcine thyroglobulin (TG) in complete (orange) and incomplete (blue) Freund's adjuvant at the indicated timepoints. Control rats were injected with saline. Plasma was collected on experimental day (Ex) 44, during the intraperitoneal glucose tolerance on embryonic day (E) 16, and on cull day E20. Animals were anaesthetised and killed on E20 via cardiac puncture, with tissues collected post‐mortem for further analyses. Original artwork by Rebecca Brady using BioRender.

TAb+ was induced by immunising rats with porcine thyroglobulin as previously reported (Hou et al., 2018). On Ex8, rats in the TAb+ group were injected subcutaneously between the scapulae with 100 µL of a 1:1 mixture of filtered porcine thyroglobulin solution (2 mg/mL, Sigma‐Aldrich #T1126) and complete Freund's adjuvant (FA) (Sigma‐Aldrich, #F5881). On Ex15, Ex22, Ex36 and Ex43, booster injections were performed by subcutaneous injection of 100 µL of porcine thyroglobulin solution in a 1:1 ratio with incomplete FA (Sigma‐Aldrich #F5506) (Fig. 1). Control rats were injected subcutaneously with 100 µL of saline at matched timepoints. Body weight, food intake, and water intake were measured daily. Vaginal impedance was monitored daily from Ex43 for at least one cycle, as defined by the appearance of two separate proestrus peaks (>4.5 × 10^3^ Ω), using the EC‐40 oestrous cycle monitor and probe (EC‐40, Fine Science Tools). Animals were mated overnight with a control male rat when in proestrus. If seminal plugs were identified in the cage the following morning, rats were regarded as pregnant, and that day was designated embryonic day (E)1. Pregnant females were monitored daily throughout gestation.

### Intraperitoneal glucose tolerance test

Random blood glucose levels were first measured prior to pregnancy at Ex44 using a glucometer (AccuCheck Performa), with a 200 µL blood sample collected via tail‐tip bleed to quantify thyroid antibodies and hormones. All animals underwent an intraperitoneal glucose tolerance test (ipGTT) at E16 (designated as *t* = −5 min) (Fig. 1). During the ipGTT, all females were fasted for 4 h before a baseline (fasting) blood glucose reading was taken. A glucose bolus was then delivered via intraperitoneal injection (1 g/kg body weight), and readings were taken at 5, 10, 15, 20, 30, 60, 90 and 120 min post‐injection. The area under the glucose curve (AUGC) was calculated as the total area under the curve from −5 to 120 min, using *y* = 0 mmol/L as the baseline. Blood was collected at several key points (fasting, 10, 30, 60, 90 and 120 min) for further analysis. For all collections, 200 µL of blood was obtained via tail‐tip bleed using heparin‐coated collection tubes, then centrifuged at 1300 *g* at 4°C. All plasma was stored at −80°C.

### Post‐mortem tissue collection

On E20, rats were heavily anaesthetised by intraperitoneal injection of a mix of ketamine/xylazine (1 ml/kg: 50 mg/mL ketamine, 10 mg/mL xylazine) (Fig. 1). When the pedal and ocular reflexes were absent, a random blood glucose reading was taken by tail‐tip bleed. Pregnant rats were then killed by cardiac puncture and removal of the heart. The whole uterus was removed and weighed, and then fetuses and placentas were removed and measured using digital callipers. Several placentas per litter were separated into junctional zone (JZ) and labyrinth zone (LAB) then weighed alongside maternal and fetal organs prior to snap‐freezing for storage at −80°C. Maternal thyroids, livers and a subset of placentas randomly selected within each litter were immersion‐fixed in 4% paraformaldehyde for 48 h. Fetuses were weighed then killed by decapitation prior to blood and tissue collection. Tail tips were collected post‐mortem from all fetuses for sex determination as described previously (Hofstee et al., 2020).

### Measurement of maternal thyroid parameters

To confirm that TAb+ was induced by treatment prior to pregnancy, TgAbs (MyBioSource, #EIA0543r) were measured in heparin anticoagulated plasma at Ex44 (29 days after first booster) using enzyme‐linked immunosorbent assays (ELISA). To confirm TAb+ status continued in late pregnancy, TgAbs were also measured at E20 (53–66 days after first booster). Samples were diluted 1:7, with pre‐pregnant samples and pregnant samples analysed on separate plates. The intra‐assay coefficient of variation was 15.6%. Samples from pregnant animals were repeat tested on a second plate and values averaged. Maternal TPOAb (Cusabio, #CSB‐E11199r) and total immunoglobulin G, A and M (IgG, Invitrogen, #88‐50490; IgA, Invitrogen, #88‐50480; IgM, Invitrogen, #88‐50540) concentrations were also assessed in EDTA anticoagulated plasma at E20 using ELISAs. The intra‐assay coefficient of variation was 3.4% for TPOAb, 3.8% for total IgG, 17.7% for total IgA and 19.0% for total IgM. All samples were run in duplicate in accordance with the manufacturer's instructions. For TPOAb, values measuring above the limit of detection were assigned the value of the top standard (80 IU/mL).

To assess whether treatment impacted thyroid hormone homeostasis, T4 and TSH were assessed in heparin‐treated plasma in late pregnancy at E20 using commercially available ELISAs (T4: Invitrogen, #EIAT4C, TSH: Demeditec Diagnostics GmbH, #DEV9977). TSH was also measured pre‐pregnancy, at Ex44. All samples were run in duplicate according to the manufacturer's instructions, with plasma diluted 1:5 for T4 analysis. The intra‐assay coefficient of variation was 3.7% for T4 and 7.8% for TSH.

### Liquid chromatography–tandem mass‐spectrometry (LC–MS/MS)

As measurements by ELISA are known to be susceptible to interference by endogenous antibodies (D'Aurizio et al., 2023), plasma T4 as well as other key hormones were measured using LC–MS/MS (Shimadzu Nexera XR LC (Shimadzu, Kyoto, Japan), coupled to a SCIEX 6500 Triple‐Quad system (MS/MS; SCIEX, Framingham, MA, USA)) using an adapted protocol (Lock et al., 2023; McBride et al., 2020). Plasma samples underwent liquid–liquid extraction using acetonitrile and ethyl acetate as previously described (Meakin et al., 2024). Briefly, to a 100 µL plasma aliquot, 20 µL deuterium‐labelled internal standards (10 µg/mL solution of cortisol‐d4 and 6β‐hydroxytestosterone‐d3, Toronto Research Chemicals, Toronto, Canada), 100 µL Milli‐Q water, and 300 µL acetonitrile were added. Solutions were vortexed at 370 *g* for 1 min, centrifuged at 9270 *g* for 10 min at 4°C, and supernatant transferred to a fresh Eppendorf tube. The remaining pellet was re‐extracted in 300 µL ethyl acetate, vortexed at 370 *g* for 1 min, centrifuged at 9270 *g* for 10 min at 4°C, and the supernatant transferred and mixed with the acetonitrile extract. Combined extracts were evaporated to dryness (Labconco Centrivap) and then derivatised with 2‐hydrazinopyridine (2‐HP) at 60°C for 20 min, using an adapted protocol (Bai et al., 2020). Derivatised samples were evaporated to dryness (Labconco Centrivap), reconstituted in 20% acetonitrile, and then injected onto an ACQUITY UPLC BEH C18 Column 130Å, 1.7 µm, 2.1 mm × 100 mm (Waters Corp, Massachusetts, US) and analytes were resolved and detected as previously described (Lock et al., 2023; McBride et al., 2020). Derivatised hormone concentrations were calculated via integration with a standard curve that ranged from 0.05 ng/mL to 100 ng/mL.

### Measurement of maternal and fetal metabolic parameters

Plasma insulin concentrations were measured in samples from the ipGTT (fasting, 10, 30, 60, 90, 120 min) as well as maternal and fetal E20 plasma via ELISA (Crystal Chem, #90060). Fetal samples were pooled for each litter. The intra‐assay coefficient of variation was 4.2%. Insulin values from the ipGTT were used to calculate area under the insulin curve (AUIC) from −5 to 120 min, using *y* = 0 µU/mL as the baseline. Homeostasis model assessment of insulin resistance (HOMA‐IR) was calculated using the equation: fasting plasma insulin (µU/mL) × fasting plasma glucose (mg/dL) ÷ 405 (Aref et al., 2013).

Several other parameters related to metabolism were quantified in maternal and fetal plasma using commercially available kits. Rat placental lactogen (rPL) was measured in maternal plasma at E16 and E20 (My BioSource, #MBS720834). Samples were diluted 1:4 and tested in duplicate across a single plate, with an intra‐assay coefficient of variation of 3.0%. Plasma triglycerides (TGs) and free fatty acids (FFAs) were also assessed in maternal and fetal E20 samples (TG: Sapphire Bioscience, Cat #990‐87445, FFAs: Sigma‐Aldrich, Cat #MAK044). The liver enzymes aspartate aminotransferase (AST) and alanine aminotransferase (ALT) were measured in maternal plasma at E20 (AST: Abcam, #ab263883, ALT: Abcam, #ab234579). All samples were assayed in duplicate across one plate in accordance with the manufacturer's instructions. The intra‐assay coefficient of variation was 1.0% for TGs, 2.5% for FFAs, 3.0% for AST and 2.9% for ALT.

Endothelin‐1 (ET‐1) concentrations were assessed in late pregnancy to determine whether TAb+ could alter markers of vasoconstriction. ET‐1 was measured in maternal EDTA treated plasma at E20 using commercially available ELISA (R&D Systems, #DET100). Samples were tested in duplicate across a single plate, with the intra‐assay coefficient of variation at 2.1%.

### Histological analysis of maternal tissues

Fixed whole thyroid glands with trachea intact were paraffin‐embedded and sectioned at 7 µm thickness. Sections containing both the left and right lobes of the thyroid gland were stained with haematoxylin and eosin and assessed for frank inflammation by an independent expert (H.B‐O). Separate sections were also stained for apoptosis using the ApopTag Peroxidase In Situ Apoptosis Detection kit (EMD Millipore, #S7100). A protocol for paraffin‐embedded tissue was followed in accordance with the kit manual, and sections were counterstained with 0.5% methyl green. Slides were imaged at 20× using the Aperio XT Brightfield Slide Scanner (Leica Biosystems). Negative controls were performed by following the TUNEL protocol but without the addition of the diaminobenzidine (DAB) substrate. The number of stained and unstained nuclei was counted across two fields, with apoptotic index determined as the percentage of positive‐stained cells per 1000 thyrocytes observed.

Paraffin‐embedded maternal livers were sectioned at 5 µm thickness and stained for collagen deposition and evidence of fibrosis using a Masson's trichrome staining protocol. Slides were imaged using the Aperio XT Brightfield Slide Scanner (Leica Biosystems) at 40× resolution and analysed in Aperio ImageScope. All analysis was conducted blind to treatment group. To evaluate the level of fibrosis in the liver, the semi‐quantitative Knodell Scoring system was used as previously published (Sant'Anna et al., 2011). Images were coded prior to the grid feature in Aperio ImageScope being used to divide the section into 6 × 4 square areas (3 × 2 mm areas) at 1× magnification. Three of those areas were randomly chosen from each section so that six areas could be evaluated per sample. Areas were then evaluated against the Knodell Scoring system, using representative images as a guide, before the mean score assigned to that tissue sample was used for analysis.

Paraffin‐embedded maternal aortas were oriented to section through the transverse plane at 5 µm thickness. Collagen content was determined using Masson's trichrome staining and elastin using Verhoeff—Van Gieson staining. Slides were imaged using the Aperio XT Brightfield Slide Scanner (Leica Biosystems) at 40× resolution. All histological analyses were blinded to treatment group. The thickness of arterial walls was measured from five random points per section across two non‐consecutive sections per aorta using Aperio ImageScope (Leica Biosystems). Aortas stained with Masson's trichrome were used to measure collagen area relative to total area using FIJI software. Elastin content was quantified in ImageScope by counting the number of elastin fibres in four random areas per aorta.

### Histological analysis of placental area and blood space

Fixed placentas were cut along the midline, paraffin‐embedded and sectioned at 6 µm thickness. A biotinylated Isolectin B4 antibody (Sigma‐Aldrich, #L2140, RRID:AB_2313663) was used to visualise fetal endothelial cells. Following dewaxing and rehydration, slides were blocked for endogenous peroxidase activity with 3% H_2_O_2_ prior to incubating with Isolectin B4 (1:35 dilution) for 2 h at room temperature. Slides were incubated with Vectastain avidin‐biotin complex, then DAB, before counterstaining with haematoxylin. Mounted sections were imaged at 40× using a Zeiss AxioScan Z1 Fluorescent Imager (Zeiss, Germany). These sections were used to measure placental zone area and total blood space. The total cross‐sectional area of JZ and LAB was measured by a blinded assessor, following the protocol previously published in our lab (Kent et al., 2023). Total blood space (combined maternal and fetal blood space area) was measured using an ImageJ macro. Briefly, three to four snapshots were taken from sections at two separate placental depths and analysed using the H&E Colour Deconvolution plugin on ImageJ. Batch analysis occurred for all sections and average values were taken for each placenta, with threshold levels kept consistent.

### Placental glycogen content

Whole JZs and LABs were crushed under liquid nitrogen and 50 mg of tissue was homogenised in ethanol extract solution (65% ethanol/10% PBS/25% H_2_O), as previously described (Cruz & Dienel, 2002). Samples were centrifuged (3820 *g*, 10 min, 4°C) then pellets were dissolved in 0.03 M HCl and incubated at 100°C for 45 min. Two aliquots of dissolved pellet samples were neutralised by the addition of 0.1 M sodium acetate, and either amyloglucosidase (AG+) or equal volume of distilled water (AG‐) was added. Both aliquots were incubated at 37°C for 2 h. Samples were centrifuged (23,900 *g*, 15 min, 24°C) and supernatants were assayed in duplicate following addition of glucose hexokinase reagent (ThermoScientific Cat# 981779). Absorbance was measured at 340 nm. Final glycogen content was derived from a glucose standard curve and calculated as the difference between the AG+ and AG‐ fraction. The intra‐assay coefficient of variation was 3.9%.

### RNA extraction, cDNA synthesis and qPCR

Total RNA was extracted from maternal liver and a representative male and female placental JZ and LAB using the RNeasy Mini Kit (Qiagen, #74104) according to the manufacturer's instructions, with 20 mg tissue used per sample. Five hundred nanogrammes of total RNA was reverse transcribed into cDNA using the iScript Reverse Transcription Supermix for qPCR (Bio‐Rad, #1725034). qPCR was performed using Fast SYBR Green Master Mix and SYBR Green primer pairs (Sigma, see Table 1). Reference genes were stably expressed across all samples. Geometric means of two chosen control genes determined gene expression using the 2^−ΔΔCT^ method, with tissue‐specific control genes detailed in the respective figure legend. Fold‐change was expressed relative to the control male group (Bustin et al., 2009; Hellemans et al., 2007).

**Table 1:** Quantitative polymerase chain reaction primer list

Group	Gene name	Gene acronym	Primer sequence
Housekeeper	Beta‐actin	*Actb*	F’ GACGACATGGAGAAAATCTG R’ ATGATCTGGGTCATCTTCTC
Housekeeper	Beta‐2‐microglobulin	*B2m*	F'ACTGGTCTTTCTACATCCTG R'AGATGATTCAGAGCTCCATAG
Housekeeper	Ribosomal protein L19	*Rpl19*	F’ CTTAGGCTACAGAAGAGGC R’ ATCTGTTGACGAGAGTTGG
Housekeeper	TATA‐box binding protein	*Tbp1*	F’ GCTAGCTAGCTTGCTAGTAC R’ CGTGCGATGCTACTAGC
Housekeeper	Ubiquitin coding gene	*Ubc*	F’ TGACAATGCAGATCTTTGTG R’ ACTCCTTCTGGATGTTGTAG
Cell growth	Insulin‐like growth factor 2	*Igf2*	F'AACCAAACAGGTTTAAGCGC R’ CTCCCAGTCGACTGCTTCCAC
Detoxification	ATP‐binding cassette, sub‐family member 2	*Abcc2*	F’ CATGGACAGTGACAAGATAATG R’ AAACTGTTCTTATAGTCCGC
Detoxification	Uridine diphosphate‐glucuronosyl‐tranferase 1a1	*Ugt1a*	F’ CTTTGTGAAAGATTACCCCAG R’ GACATAGGCTTCAAATTCCTG
Glucose metabolism	Glycogen synthase kinase 3 beta	*Gsk3b*	F’ ACTCAAGAACTGTCAAGTAAC R’ TCCAGCATTAGTATCTGAGG
Glucose metabolism	Solute carrier family 2‐member 1	*Slc2a1*	F’ CAATATGTGGAGCAACTGTG R’ AGTAGGTGAAGATGAAGAAGAG
Glucose metabolism	Solute carrier family 2‐member 2	*Slc2a2*	F’ GTGTTGCTGGATAAGTTCAC R’ CTCAAAGAAACTGACGAAGAG
Glucose metabolism	Solute carrier family 2‐member 3	*Slc2a3*	F’ TTCACTGTAGTCTCTCTGTTC R’ GGCTTCATATTCATCCTTCAG
Immune function	Interleukin‐1‐β	*Il1b*	F’ TAAGCCAACAAGTGGTATTC R’ AGGTATAGATTCTTCCCCTTG
Immune function	Tumour necrosis factor α	*Tnfa*	F‐5’‐GAAACACACGAGACGCTGAA R ‐5’‐GAAAGCCCATTGGAATCCTT
Lipid transporters	Platelet glycoprotein 4	*Cd36*	F’ AAGGAATTTGTCCTATTGGG R’ GAGACTTCTCAACAAAAGGTG
Placental lactogen	Prolactin‐3B1	*Prl3b1*	F’ CTACACTCATGATCTTGCTTC R’ GCCAGGCTTGTAAAATAGTG
Placental lactogen	Prolactin‐3D1	*Prl3d1*	F’ AGACCTTATACAACAGGACTC R’ ATGGCAAAAAGATGAGTGTC
Vascularisation	Binding immunoglobulin protein	*Hspa5*	F’ GATCATCAATGAGCCAACAG R’ ATTGTCAATGGTGAGAAGAG
Vascularisation	Soluble fms‐like receptor tyrosine kinase 1	*Flt1*	F'CCAGAAGTCGTATGGTTAAAAG R'GCTGTGAGGTTTCTAAATAGC
Vascularisation	Kinase insert domain receptor	*Kdr*	F’ AAACTGGATAAAATGGGCG R’ AGCCTTTTAGGTAGAGTCAG
Vascularisation	Platelet‐derived growth factor subunit β	*Pdgfb*	F’ AAAGATCGAAATTGTGAGAAAG R’ CGAGTTTGAGGTGTCTTG
Vascularisation	Transforming growth factor β 1	*Tgfb1*	F’ GGAAATCAATGGGATCAGTC R’ CTGAAGCAGTAGTTGGTATC
Vascularisation	Vascular endothelial growth factor subunit a	*Vegfa*	F’ GATAGAGTATATCTTCAAGCCG R’ CTCATCTCTCCTATGTGCTG
Vasoconstrictor	Endothelin 1	*Edn1*	F’ GAAAACTAAGAAGGTTGGAGG R’ TGTATCAACTTCTGGTCTCTG
Vasoconstrictor	Prostaglandin synthase 2	*Ptgs2*	F’ CTCATACTGATAGGAGAGACG R’ TCGAACTTGAGTTTGAAGTG

### Statistical analysis

All data were analysed using GraphPad Prism v8.3.1 (GraphPad Software, RRID:SCR_002798). All data were assessed for normality using a Shapiro–Wilk test, with *P* > 0.05 accepted as normally distributed. If data followed a normal distribution, they were analysed using an unpaired *t* test with Welch's correction. If data did not follow a normal distribution, they were analysed using a Mann–Whitney U test (P^M^). Fetal and placental data that were split by sex, were analysed using a two‐way ANOVA with Sidak's multiple comparisons test. All data are expressed as means ± SD with *P* < 0.05 considered statistically significant.

One control animal was excluded from all analyses as it was not pregnant at the time of killing. Two animals from the control group and one from the TAb+ group were excluded from all maternal, placental and fetal analysis due to abnormally low litter size (four pups or less), but are still represented in the litter size analysis. As a result, the final sample sizes for pre‐pregnancy data analyses were *n* = 9 for the control group and *n* = 10 for the TAb+ group. The final sample sizes for maternal, fetal and placental data analysis were *n* = 7 for the control group and *n* = 9 for the TAb+ group. Additionally, ipGTT was not performed in one control animal due to COVID lockdown, and one control and one treated animal did not respond to the glucose injection at the time of the ipGTT. The animals that did not respond were excluded from all time course analyses (ipGTT and insulin) but included for the fasting analyses (HOMA‐IR). Thus, the final sample sizes were *n* = 5–7 for the control group and *n* = 7–9 for the TAb+ group.

## Results

### The effect of TAb+ on maternal thyroid function

**Figure 2:**
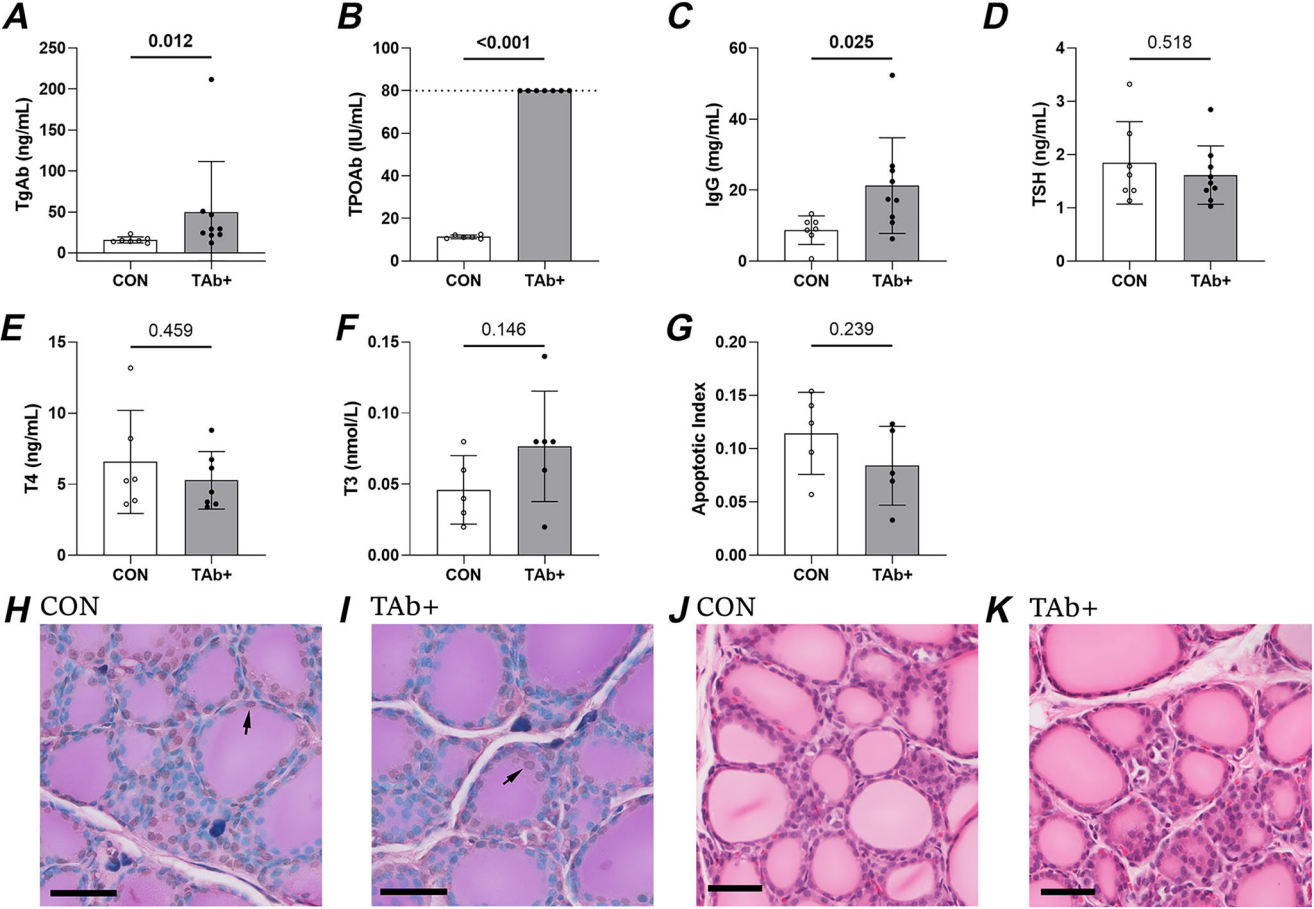
Comparison of thyroid hormones, antibodies and morphology at late pregnancy in control and TAb+ rats Maternal plasma concentrations of thyroglobulin antibodies (TgAb, ng/mL) (*A*), thyroid peroxidase antibodies (TPOAb, IU/mL) (*B*), total immunoglobulin G (IgG, mg/mL) (*C*), thyroid stimulating hormone (TSH, ng/mL) (*D*), thyroxine (T4, ng/mL) (*E*) and triiodothyronine (T3, nmol/L) (*F*) at embryonic day (E) 20. Dashed line in (*B*) represents upper limit of detection of the TPOAb ELISA, with all TAb+ samples above the upper limit. *E* and *F*, measured by liquid chromatography mass spectroscopy (LC–MS/MS). Histological analysis of apoptosis index in maternal thyroids collected at E20 (*G*), with representative images for TUNEL‐stained thyroids in control (*H*) and TAb+ (*I*) animals taken at 40× magnification. Representative images of control (*J*) and TAb+ (*K*) thyroids stained with H&E taken at 20× magnification. Scale bars represent 50 µm, black arrows in (*H*), and (*I*), identifying apoptotic nuclei. CON = control animals, *n* = 5–7. TAb+ = thyroid antibody positive animals, *n* = 5–9. All data are presented as means ± SD with individual point shown. Data were compared using unpaired *t* tests with Welch's correction if data were normally distributed. Non‐normally distributed data were analysed using Mann–Whitney U tests. *P* < 0.05 was considered statistically significant.

This study was designed to generate a rodent model of TAb+ to characterise the impact of TAbs on maternal, placental and fetal outcomes. TgAbs and TSH were measured prior to pregnancy (Ex44) with the limited sample obtained at this timepoint, while a complete thyroid assessment was performed in late pregnancy (E20) when greater sample volume was available. Compared with control animals, TgAbs were elevated in TAb+ animals by 69% on Ex44 (P^M^ = 0.006, Table 2) while TSH was unchanged (CON = 0.80 ± 0.15 ng/mL, TAb+ = 1.11 ± 0.40 ng/mL, *P* = 0.124). TgAbs were increased in TAb+ dams by 204% on E20 (P^M^ = 0.012, Fig. 2*A*). A higher than eightfold increase in TPOAbs was seen in treated dams at E20, with all TAb+ plasma samples higher than the upper limit of detection for the ELISA (P^M^ < 0.001, Fig. 2*B*). TAb+ resulted in a 143% increase in total IgG in late pregnancy (*P* = 0.025, Fig. 2*C*), but there were no changes to total IgA or IgM (IgA: *P* = 0.222, IgM: P^M^ = 0.999). While T4 as measured by ELISA was significantly elevated in TAb+ rats at E20 (CON = 8.665 ± 3.04 ng/mL, TAb+ = 76.14 ± 27.84 ng/mL, *P* < 0.001), there were no changes in TSH (*P* = 0.518, Fig. 2*D*). Given the known interference between TAb and ELISA measurement of thyroid hormones, we performed an additional assessment of thyroid hormone concentrations using LC–MS/MS, a method that is not sensitive to interference by endogenous anti‐thyroid antibodies. When measured by LC–MS/MS, there was no effect of TAb+ on T4 (*P* = 0.459, Fig. 2*E*) or T3 (P^M^ = 0.146, Fig. 2*F*). These results suggest that the elevated T4 concentrations seen by ELISA in this study were likely due to interference by anti‐thyroid antibodies (D'Aurizio et al., 2023). TAb+ had no impact on thyroid apoptosis (*P* = 0.239, Fig. 2*G*) as indicated by TUNEL staining (Fig. 2*H*,*I*). Additional examination of thyroid histo‐morphology by an expert veterinary pathologist confirmed no overt pathology in the thyroids from these rats (Fig. 2*J*,*K*). Given that there was no indication of frank inflammation or apoptosis within the thyroid, this would suggest a model of TAb+ with no overt changes to thyroid biology during pregnancy.

**Table 2:** Comparison of control and TAb+ maternal parameters prior to and at late pregnancy

Maternal parameter	Control (*n* = 7–9)	TAb + (*n* = 9–10)	*P* value
Body weight on Ex0 (g)	184.1 ± 7.2	176.4 ± 11.2	*P* = 0.092
Weight gain prior to pregnancy (g)	44.67 ± 7.60	37.80 ± 7.83	*P* = 0.069
TgAb concentration at Ex44 (ng/mL)	27.56 ± 2.13	46.64 ± 43.23	**P^M^ = 0.006**
Blood glucose level at Ex44 (mmol/L)	6.71 ± 0.54	6.96 ± 0.36	P^M^ = 0.080
Body weight on E0	227.7 ± 5.2	215.4 ± 11.6	*P* = **0.016**
Total weight gain in pregnancy (g)	96.43 ± 9.12	92.11 ± 5.21	*P* = 0.292
Food intake (g/gbw/day)	0.113 ± 0.003	0.114 ± 0.005	*P* = 0.500
Water intake (mL/gbw/day)	0.110 ± 0.010	0.116 ± 0.019	*P* = 0.444
Body weight on E20 (g)	324.1 ± 13.7	307.6 ± 12.7	*P* = **0.028**
Body weight on E20 (no uterus) (g)	287.1 ± 11.7	273.7 ± 11.6	*P* = **0.040**
Uterus weight (g)	37.03 ± 5.16	33.88 ± 7.33	P^M^ = 0.332
Maternal heart weight (mg)	967.6 ± 80.2	884.3 ± 55.6	**P^M^ = 0.023**
Heart weight: body weight (mg/g)	2.99 ± 0.23	2.88 ± 0.16	*P* = 0.306
Maternal liver weight (g)	10.44 ± 0.79	10.41 ± 0.59	*P* = 0.937
Liver weight: body weight (mg/g)	32.17 ± 1.47	33.83 ± 0.99	*P* = **0.028**
Pancreas weight (mg)	714.3 ± 181.1	654.8 ± 115.1	*P* = 0.466
Pancreas weight: body weight (mg/g)	2.19 ± 0.49	2.13 ± 0.38	*P* = 0.799
Spleen weight (mg)	612.6 ± 36.7	622.8 ± 38.7	*P* = 0.599
Spleen weight: body weight (mg/g)	1.89 ± 0.07	2.03 ± 0.12	*P* = **0.015**

*Note*: Significant *P* values (*P* < 0.05) are shown in bold.

Abbreviations: E, embryonic day; Ex, experimental day; *P*, unpaired *t* test with Welch's correction; P^M^, Mann–Whitney test; T4, thyroxine; TgAb, anti‐thyroglobulin antibodies; TPOAb, anti‐thyroperoxidase antibodies.

### The effect of TAb+ on maternal pregnancy parameters

**Figure 3:**
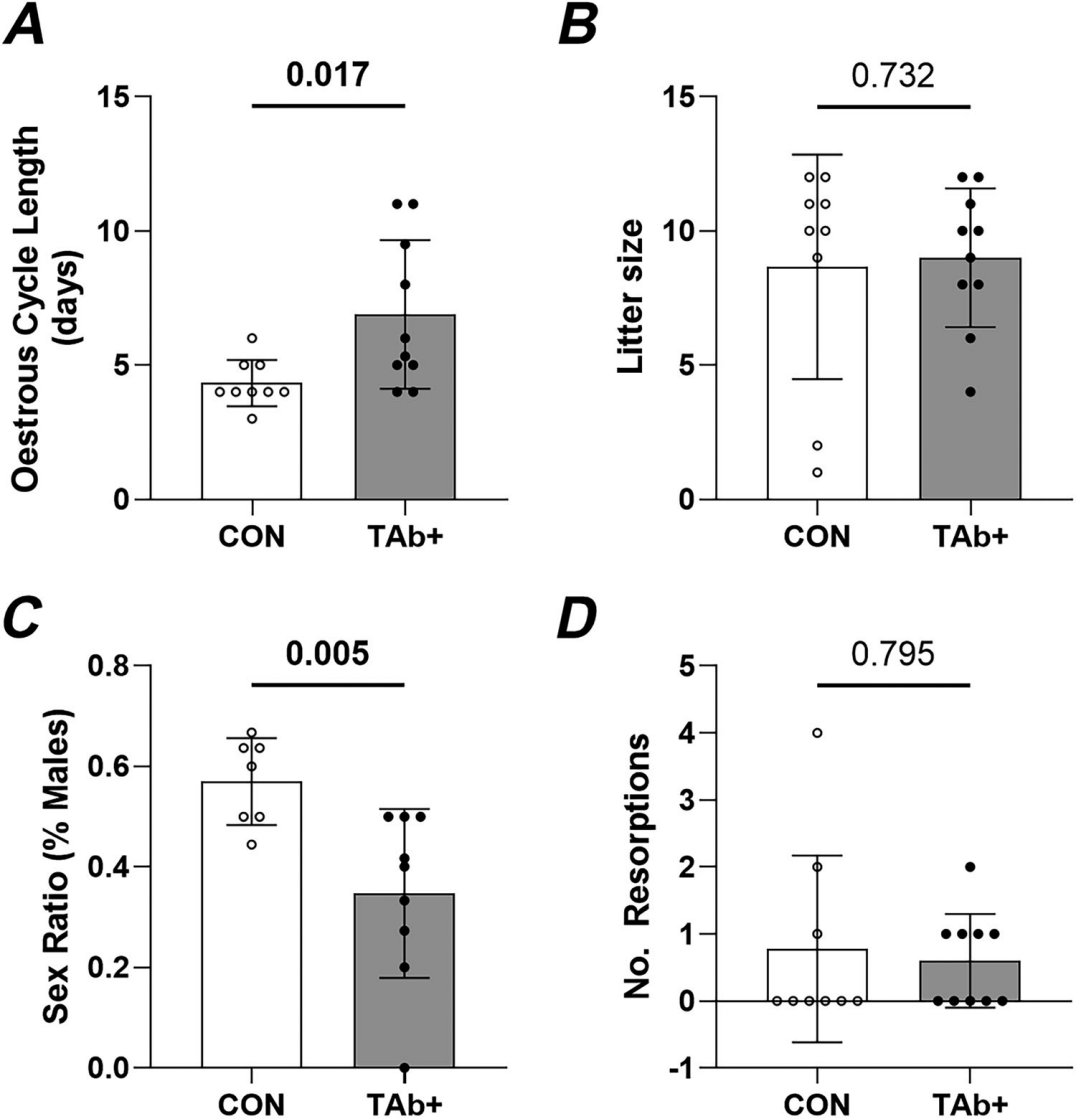
The effect of TAb+ on oestrous cycling and fertility Oestrous cycling prior to pregnancy was measured using an impedance probe, with total oestrous cycle length (days) assessed (*A*). Parameters relating to pregnancy success and fertility are shown in (*B*)–(*D*), with litter size (*B*), sex ratio (% males) (*C*) and number of resorptions (*D*), all quantified at the end of pregnancy (embryonic day 20). CON = control animals, *n* = 7–9. TAb+ = thyroid antibody positive animals, *n* = 9–10. Data in (*A*), presented as ratio of animals cycling normally to those cycling abnormally, with comparisons between CON and TAb+ made using Fisher's exact test. Data in (*B*)–(*E*) are presented as means ± SD with individual points shown and were compared using unpaired *t* tests with Welch's correction if data were normally distributed. Non‐normally distributed data were analysed using Mann–Whitney U tests. *P* < 0.05 was considered statistically significant.

Neither body weight at the start of the protocol (*P* = 0.092, Table 2) nor weight gained prior to pregnancy (*P* = 0.069, Table 2) were significantly different between controls and TAb+ dams. Body weight on E0 was on average 12.3 g less in TAb+ dams compared with control dams (P^M^ = 0.016, Table 2). Oestrous cycle length was, on average, 3 days longer in dams with TAb+ compared with controls (*P* = 0.017, Fig. 3*A*). There was no difference in total weight gain throughout pregnancy (*P* = 0.292, Table 2), nor were there any differences in daily chow (*P* = 0.500, Table 2) or water intake (*P* = 0.444, Table 2). On E20, maternal body weight was, on average, 16.5 g less in TAb+ dams compared with control dams prior to removal of the uterus (*P* = 0.028, Table 2). Maternal body weight remained lower in the TAb+ dams following removal of the uterus (13.4 g difference, *P* = 0.040, Table 2). Neither the uterus weight (*P* = 0.332, Table 2) nor litter size (P^M^ = 0.732, Fig. 3*B*) was impacted by treatment. Interestingly, the proportion of males relative to total litter size was reduced in the TAb+ group (*P* = 0.005, Fig. 3*C*), but there was no difference in number of resorptions at E20 (P^M^ = 0.795, Fig. 3*D*). Maternal heart weight was reduced in TAb+ dams compared with controls (*P* = 0.023, Table 2), but this effect was no longer present following normalisation to body weight (*P* = 0.306, Table 2). In contrast, whole liver weight was not significantly affected by treatment in TAb+ dams (*P* = 0.937, Table 2) but was significantly increased following normalisation to body weight (*P* = 0.028, Table 2). Maternal TAb+ did not affect pancreas weight (*P* = 0.466, Table 2), normalised pancreas weight (*P* = 0.799, Table 2) or any other maternal organ weights (Table 2).

### The effect of TAb+ on glucose homeostasis and metabolism in pregnancy

**Figure 4:**
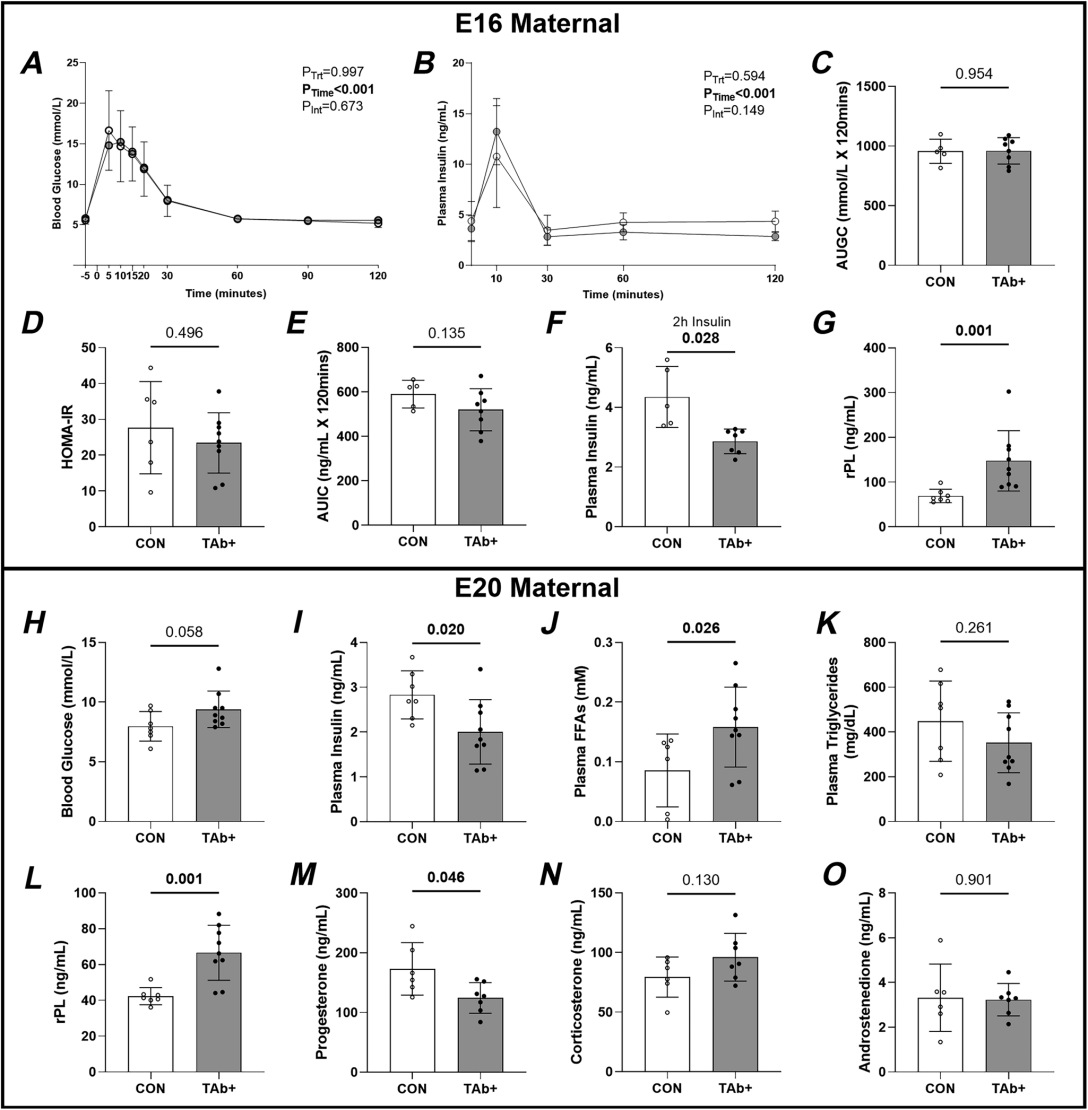
The effect of TAb+ on maternal metabolism in late pregnancy Maternal blood glucose levels (BGLs, mmol/L) (*A*) and insulin levels (ng/mL) (*B*) throughout the intraperitoneal glucose tolerance test (ipGTT), area under the glucose curve (AUGC) (*C*), homeostatic model of insulin resistance (HOMA‐IR) (*D*), area under the insulin curve (AUIC) (*E*), 2 h plasma insulin (ng/mL) (*F*) and rat placental lactogen (rPL, ng/mL) (*G*) on embryonic day (E) 16. Maternal random BGLs (mmol/L) (*H*), plasma insulin levels (ng/mL) (*I*), plasma free fatty acids (FFAs, mM) (*J*), plasma triglycerides (ng/dL) (*K*), rPL (ng/mL) (*L*), progesterone (ng/mL) (*M*), corticosterone (ng/mL) (*N*) and androstenedione (ng/mL) (*O*) at E20. CON = control animals, *n* = 5–7. TAb+ = thyroid antibody positive animals, *n* = 7–9. All data are presented as means ± SD with individual points shown. Data in (*A*) and (*B*) were compared using a mixed effects model with Sidak's multiple comparisons test. Data in (*C*)–(*O*), were compared using unpaired *t* tests with Welch's correction if data were normally distributed. Non‐normally distributed data were analysed using Mann–Whitney U tests. *P* < 0.05 was considered statistically significant.

Overall, TAb+ had minimal effects on maternal glucose homeostasis during pregnancy. TAb+ did not impact blood glucose measurements during the ipGTT (PTrt = 0.997, Fig. 4*A*), plasma insulin during the ipGTT (*P* = 0.594, Fig. 4*B*) or total AUGC (*P* = 0.934, Fig. 4*C*). Both HOMA‐IR (*P* = 0.496, Fig. 4*D*) and the AUIC (*P* = 0.135, Fig. 4*E*) were not affected, although plasma insulin at the 2 h time point was 34% lower on average in TAb+ animals (*P* = 0.028, Fig. 4*F*). Placental lactogen concentrations at E16 were increased by 118% in the TAb+ group compared with controls (P^M^ = 0.001, Fig. 4*G*).

Random, non‐fasted blood glucose concentrations were not affected by TAb+ prior to pregnancy at Ex44 (P^M^ = 0.080, Table 2) or at late gestation (*P* = 0.058, Fig. 4*H*). Random insulin concentrations at E20 were 30% lower in the TAb+ group (*P* = 0.020, Fig. 4*I*). Maternal plasma FFAs were increased in TAb+ rats at E20 (*P* = 0.026, Fig. 4*J*) while plasma TGs were unaltered (*P* = 0.261, Fig. 4*K*). Placental lactogen concentrations at E20 were increased 57% by TAb+ (*P* = 0.001, Fig. 4*L*). This significant increase in rPL in TAb+ plasma remained after normalising to total placenta weight, with TAb+ placentas still producing, on average, 11.4 ng/mL more rPL per gramme of placenta compared with control placentas (*P* = 0.023). TAb+ during pregnancy resulted in a 28% decrease in plasma progesterone (*P* = 0.046, Fig. 4*M*), with no changes to plasma corticosterone (*P* = 0.130, Fig. 4*N*) or androstenedione (*P* = 0.901, Fig. 4*O*).

### The effect of TAb+ on maternal liver and vascular biology

**Figure 5:**
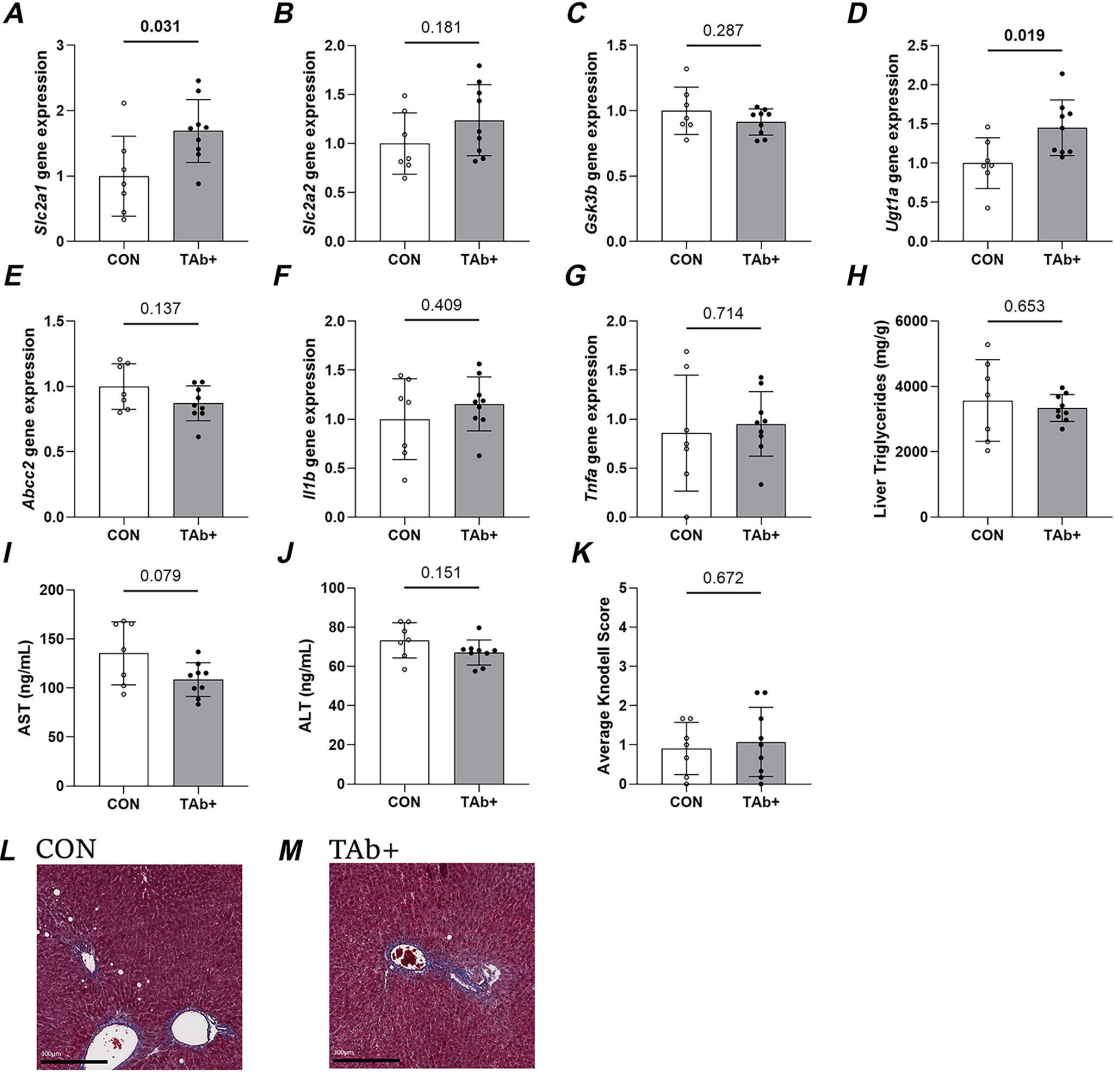
The effect of TAb+ on maternal liver in late pregnancy Relative expression of key genes involved in glucose metabolism, bile storage and inflammation were assessed in maternal liver, including glucose transporter 1 (Slc2a1) (*A*), glucose transporter 2 (Slc2a2) (*B*), glycogen synthase kinase 3 beta (Gsk3b) (*C*), uridine diphosphate glucuronosyltransferase (Ugt1a) (*D*), ATP‐binding cassette, sub‐family member 2 (Abcc2) (*E*), interleukin‐1‐beta (Il1b) (*F*) and tumour necrosis factor α (Tnfa) (*G*). All gene expression in (*A*)–(*G*) is normalised to the geomean of Actb, Ubc and B2m. Triglyceride concentration in maternal liver (mg/g tissue) (*H*). Maternal plasma concentration of aspartate transaminase (AST, ng/mL) (*I*) and alanine transaminase (ALT, ng/mL) (*J*). (*K)*–(*M)*, examination of histopathological representation of hepatic fibrosis using Masson's trichrome staining and analysed using the Knodell Trichrome Staining Fibrotic Scoring System (Sant'anna et al 2011). Graphical representation of average Knodell scores applied to each sample per treatment (K). Histological display of level 1 on the Knodell Scale showing fibrotic accumulation around the portal vein from a CON sample (*L*) and TAb+ sample (*M*), taken at 20× magnification with 300 µm scale bar in bottom left corner. CON = control animals, *n* = 7. TAb+ = thyroid antibody positive animals, *n* = 9. All data are presented as means ± SD with individual points shown. Data were compared using unpaired *t* tests with Welch's correction if data were normally distributed. Non‐normally distributed data were analysed using Mann–Whitney U tests. *P* < 0.05 was considered statistically significant.

As maternal liver relative to total body weight was increased in TAb+ dams, several markers of glucose transport, function, inflammation and fibrosis were examined in this tissue. TAb+ increased *Slc2a1* (*P* = 0.031, Fig. 5*A*) but not *Slc2a2* (*P* = 0.181, Fig. 5*B*) or *Gsk3b* (*P* = 0.287, Fig. 5*C*) mRNA expression. Two markers of bilirubin metabolism were also explored in maternal liver, with TAb+ leading to an increase in *Ugt1a* (*P* = 0.019, Fig. 5*D*) but not *Abcc2* (*P* = 0.137, Fig. 5*E*) mRNA expression. There was no effect of TAb+ on hepatic mRNA expression of inflammatory markers *Il1b* (*P* = 0.409, Fig. 5*F*) or *Tnfa* (*P* = 0.714, Fig. 5*G*). Hepatic triglyceride concentrations were also not impacted by TAb+ (*P* = 0.653, Fig. 5*H*). Additionally, TAb+ did not impact plasma concentrations of AST (*P* = 0.079, Fig. 5*I*) or ALT (*P* = 0.151, Fig. 5*J*). There was also no evidence of increased hepatic fibrosis (Fig. 5*K*,*L*,*M*).

**Figure 6:**
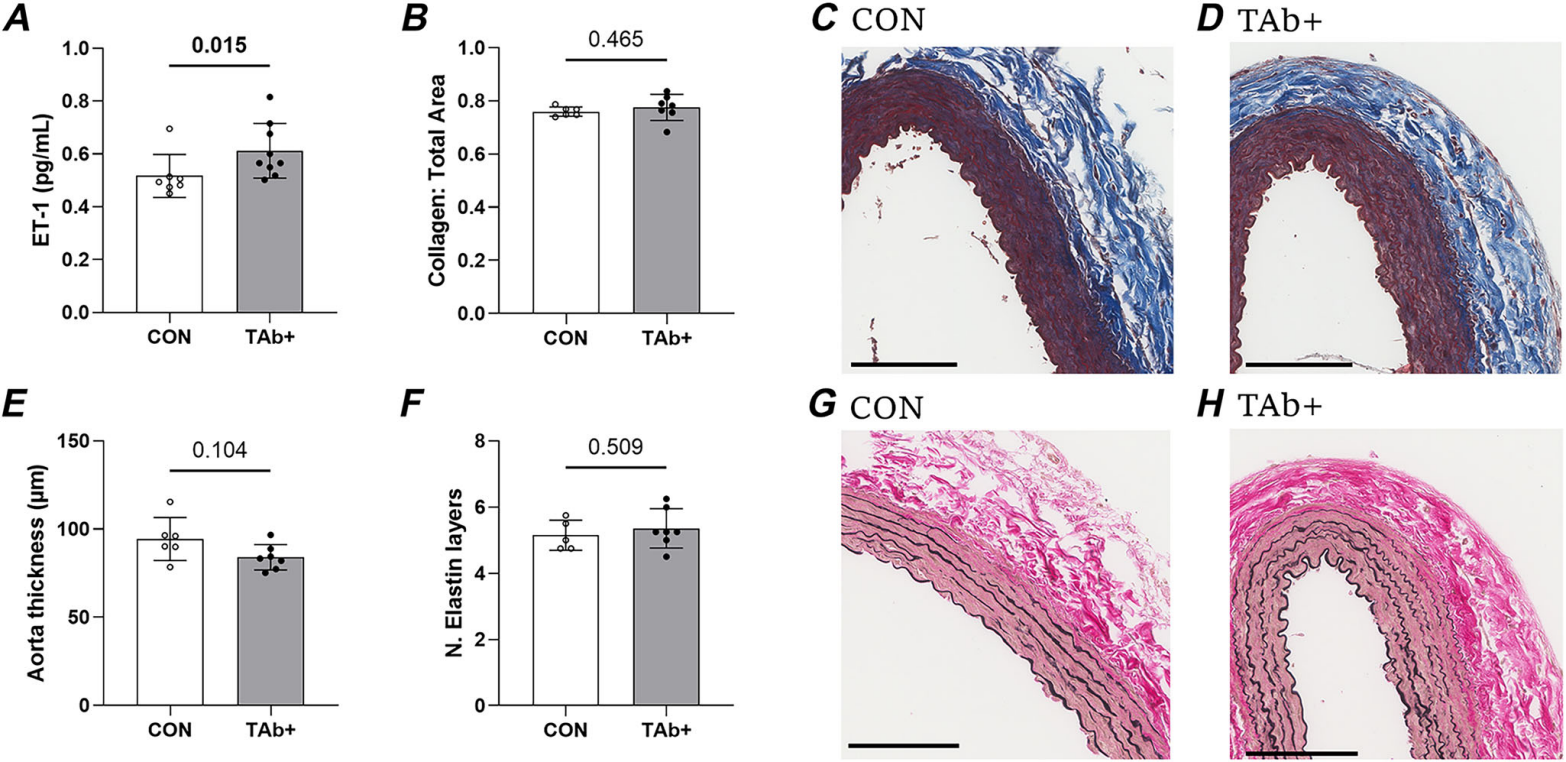
The effect of TAb+ on plasma endothelin‐1 and maternal aorta composition in late pregnancy Maternal plasma concentration of endothelin‐1 (pg/mL) (*A*). (*B)*–(*D)*, examination of histopathological representation of maternal aortic fibrosis using Masson's trichrome staining. Collagen area relative to total aorta area (*B*) with representative images for Masson's trichrome stained aortas from a control sample (*C*) and TAb+ sample (*D*). (*E)*–(*H)*, examination of morphology and elastin content in aorta using Verhoff–Van Gieson staining, with aortic thickness (µm) (*E*) and number of elastin layers (*F*). Representative images of Verhoff–Van Gieson‐stained aortas from a control sample (*G*) and TAb+ sample (*H*). Both histology images captured at 40× magnification with 150 µm scale bars indicated by a black line. CON = control animals injected with saline only, *n* = 5–7. TAb+ = thyroid antibody positive animals injected with porcine thyroglobulin and Freund's adjuvant, *n* = 7–9. All data are presented as means ± SD with individual points shown. Data were compared using unpaired *t* tests with Welch's correction if data were normally distributed. Non‐normally distributed data were analysed using Mann–Whitney U tests. *P* < 0.05 was considered statistically significant.

As TAbs have previously been suggested to influence aortic VSMCs (Zhang et al., 2019), the impact of TAb+ on select markers of vascular biology were also explored. There was an 18% increase in maternal plasma ET‐1 in TAb+ dams (P^M^ = 0.015, Fig. 6*A*), but there was no impact on collagen content (*P* = 0.465, Fig. 6*B*–*D*), thickness (*P* = 0.103, Fig. 6*E*) or elastin content (*P* = 0.509, Fig. 6*F*–*H*) in maternal aortas at E20.

### The effect of TAb+ on placental and fetal outcomes

**Figure 7:**
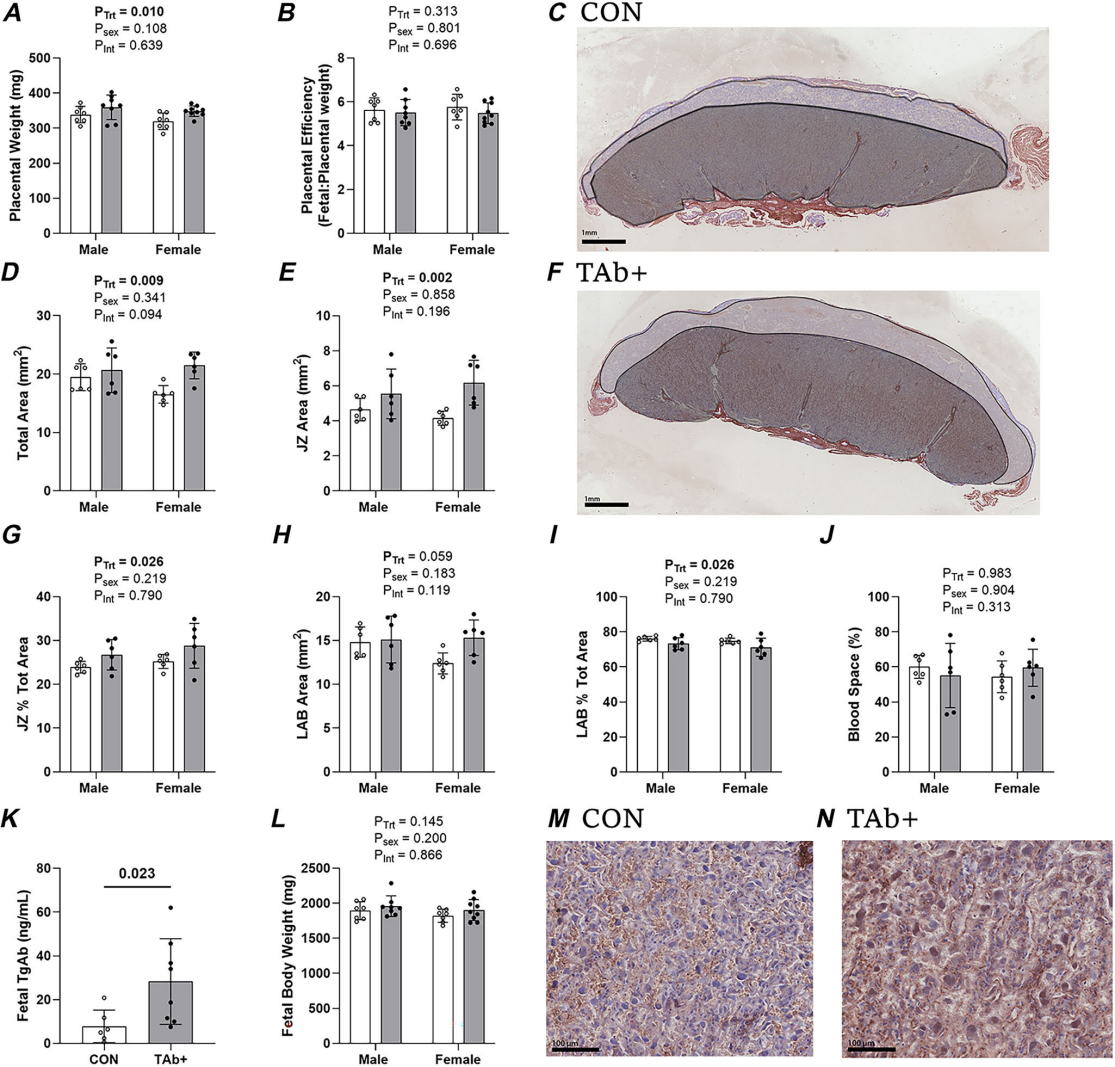
The effect of maternal TAb+ on fetal and placental morphology Analysis of placental morphology at embryonic day 20, with placental weight (mg) (*A*), placental efficiency (fetal weight: placental weight) (*B*), total area (mm^2^) (*D*), total junctional zone (JZ) area (mm^2^) (*E*), JZ as % total area (*G*), total labyrinth (LAB) area (mm^2^) (*H*), LAB as % total area (*I*) and total blood space area (*J*). Placental area measures were taken from two non‐consecutive placental sections and averaged, with a representative image of a male placental section with JZ (light) and LAB (dark) highlighted in a control (*C*) and TAb+ (*F*) sample. Scale bar (1 mm) indicated in bottom left corner. Total blood space quantified using an ImageJ macro, with a representative image of a male placental section in a control (*M*) and TAb+ (*N*) sample. Scale bar (100 µm) indicated in bottom left corner. Pooled fetal plasma samples were assessed for thyroglobulin antibodies (TgAb, ng/mL) (*K*). Fetuses were weighed at time of cull, on embryonic day 20, with fetal weight (mg) shown in (*L*). Pups and placentas from control dams indicated by white bars, *n* = 6–7/sex. Pups and placentas from TAb+ dams indicated by grey bars, *n* = 6–9/sex. All data are presented as scatter plots with means ± SD represented. Data points in (*A*), (*B*), (*K*) and (*L*) represent averaged value for the whole litter. Data points in (*D*)–(*J*) are representative male and female samples from each litter. All data except for (*K*) analysed using two‐way ANOVA with treatment and sex as factors and Sidak's multiple comparisons test (pairwise within sex). Data in (*K*) analysed by Student's unpaired *t* test with Welch's correction. *P* < 0.05 was considered statistically significant.

Placenta weight was increased due to maternal TAb+ (P_Trt_ = 0.010, Fig. 7*A*), although placental efficiency was not affected (P_Trt_ = 0.313, Fig. 7*B*). This increase was seen in placental JZ weight (P_Trt_ = 0.022, Table 3), but not placental LAB weight (P_Trt_ = 0.053, Table 3). LAB weight (P_Sex_ = 0.001, Table 3) and the JZ:LAB weight ratio (P_Sex_ = 0.016, Table 3) were higher in males than females. Maternal TAb+ had no impact on placental dimensions when measured using callipers (Table 3), but histological analysis of placental morphology showed TAb+ increased total placental cross‐sectional area (P_Trt_ = 0.009, Fig. 7*D*), with this increase seen in net JZ area (P_Trt_ = 0.002, Fig. 7*E*). Placental JZs from TAb+ litters were 13% larger than control JZs relative to total placental area (P_Trt_ = 0.026, Fig. 7*G*). Net placental LAB area was not affected by TAb+ (P_Trt_ = 0.059, Fig. 7*H*) but TAb+ exposed LABs were 5% smaller than control LABs relative to total placental area (P_Trt_ = 0.026, Fig. 7*I*). Total blood space area (maternal plus fetal blood spaces) was not affected by maternal TAb+ (P_Trt_ = 0.983, Fig. 7*J*).

**Table 3:** Comparison of control and TAb+ placental and fetal weight and dimensions at E20

Parameter	Males		Females		*P* value
	Control (*n* = 7)	TAb + (*n* = 9)	Control (*n* = 7)	TAb + (*n* = 10)	
Placenta length (mm)	12.62 ± 0.64	12.89 ± 0.68	12.50 ± 0.46	13.10 ± 0.97	P_Trt_ = 0.088 P_Sex_ = 0.811 P_Int_ = 0.466
Placenta width (mm)	10.88 ± 0.56	11.38 ± 0.84	11.00 ± 0.47	11.41 ± 0.57	P_Trt_ = 0.054 P_Sex_ = 0.735 P_Int_ = 0.846
Placental length‐to‐width ratio (mm/mm)	1.16 ± 0.05	1.14 ± 0.06	1.14 ± 0.04	1.15 ± 0.04	P_Trt_ = 0.633 P_Sex_ = 0.743 P_Int_ = 0.248
Placenta depth (mm)	3.58 ± 0.22	3.49 ± 0.32	3.33 ± 0.39	3.45 ± 0.23	P_Trt_ = 0.881 P_Sex_ = 0.177 P_Int_ = 0.307
Junctional zone weight (JZ, mg)	116.8 ± 20.3	120.8 ± 19.3	109.9 ± 14.8	135.5 ± 12.8	**P_Trt_ = 0.022** P_Sex_ = 0.531 P_Int_ = 0.090
Labyrinth zone weight (LAB, mg)	189.6 ± 16.2	199.2 ± 18.2	167.0 ± 12.6	180.2 ± 14.9	P_Trt_ = 0.053 **P_Sex_ = 0.001** P_Int_ = 0.755
JZ weight: LAB weight	0.629 ± 0.097	0.610 ± 0.114	0.664 ± 0.110	0.763 ± 0.086	P_Trt_ = 0.290 **P_Sex_ = 0.016** P_Int_ = 0.117
Crown‐rump length (mm)	26.85 ± 1.23	27.68 ± 1.08	27.40 ± 0.68	26.93 ± 1.20	P_Trt_ = 0.662 P_Sex_ = 0.799 P_Int_ = 0. 108
Head length (mm)	11.40 ± 0.33	11.59 ± 0.36	11.10 ± 0.37	11.37 ± 0.47	P_Trt_ = 0.114 P_Sex_ = 0.079 P_Int_ = 0.794
Head width (mm)	7.29 ± 0.28	7.51 ± 0.37	7.29 ± 0.25	7.24 ± 0.23	P_Trt_ = 0.419 P_Sex_ = 0.227 P_Int_ = 0.229
Head length‐to‐width ratio (mm/mm)	1.57 ± 0.04	1.55 ± 0.06	1.52 ± 0.05	1.57 ± 0.04	P_Trt_ = 0.435 P_Sex_ = 0.555 P_Int_ = 0.079
Hind limb length (mm)	5.73 ± 0.73	6.03 ± 0.43	5.94 ± 0.62	5.84 ± 0.35	P_Trt_ = 0.609 P_Sex_ = 0.958 P_Int_ = 0.295
Brain weight (mg)	107.1 ± 4.2	112.0 ± 6.9	104.2 ± 7.6	109.8 ± 6.7	**P_Trt_ = 0.032** P_Sex_ = 0.281 P_Int_ = 0. 890
Brain (mg): body weight (g)	58.59 ± 5.87	55.46 ± 5.15	57.39 ± 2.56	58.78 ± 4.03	P_Trt_ = 0.594 P_Sex_ = 0.515 P_Int_ = 0.171
Heart weight (mg)	9.53 ± 1.36	10.51 ± 1.57	9.88 ± 1.72	10.87 ± 0.62	P_Trt_ = 0.054 P_Sex_ = 0.474 P_Int_ = 0.990
Heart (mg): body weight (g)	5.19 ± 0.86	5.28 ± 0.37	5.45 ± 0.95	5.88 ± 0.53	P_Trt_ = 0.318 P_Sex_ = 0.100 P_Int_ = 0.499
Liver weight (mg)	128.8 ± 17.9	143.1 ± 20.8	123.0 ± 13.0	126.8 ± 15.3	P_Trt_ = 0.150 P_Sex_ = 0.084 P_Int_ = 0.400
Liver (mg): body weight (g)	68.54 ± 3.29	72.71 ± 7.90	67.40 ± 3.31	69.78 ± 7.02	P_Trt_ = 0.138 P_Sex_ = 0.351 P_Int_ = 0.680

*Note*: Data are presented as means ± SD. Data were analysed using a two‐way ANOVA with Sidak's multiple comparisons test. Significant *P* values (*P* < 0.05) are shown in bold.

Maternal TAb+ resulted in a 261% increase in fetal TgAbs (*P* = 0.023, Fig. 7*K*) but did not alter fetal body weight (P_Trt_ = 0.145, Fig. 7*L*) or any other fetal dimensions (Table 3). TPOAbs were not measured in fetal plasma due to limited sample volume. Fetal brain weight (P_Trt_ = 0.032, Table 3) was significantly elevated in fetuses from TAb+ dams, but no difference was observed once normalised to total body weight (*P* = 0.916, Table 3). TAb+ had no impact on the weight of any other fetal organs examined (Table 3).

### The effect of maternal TAb+ on placental and fetal metabolism

**Figure 8:**
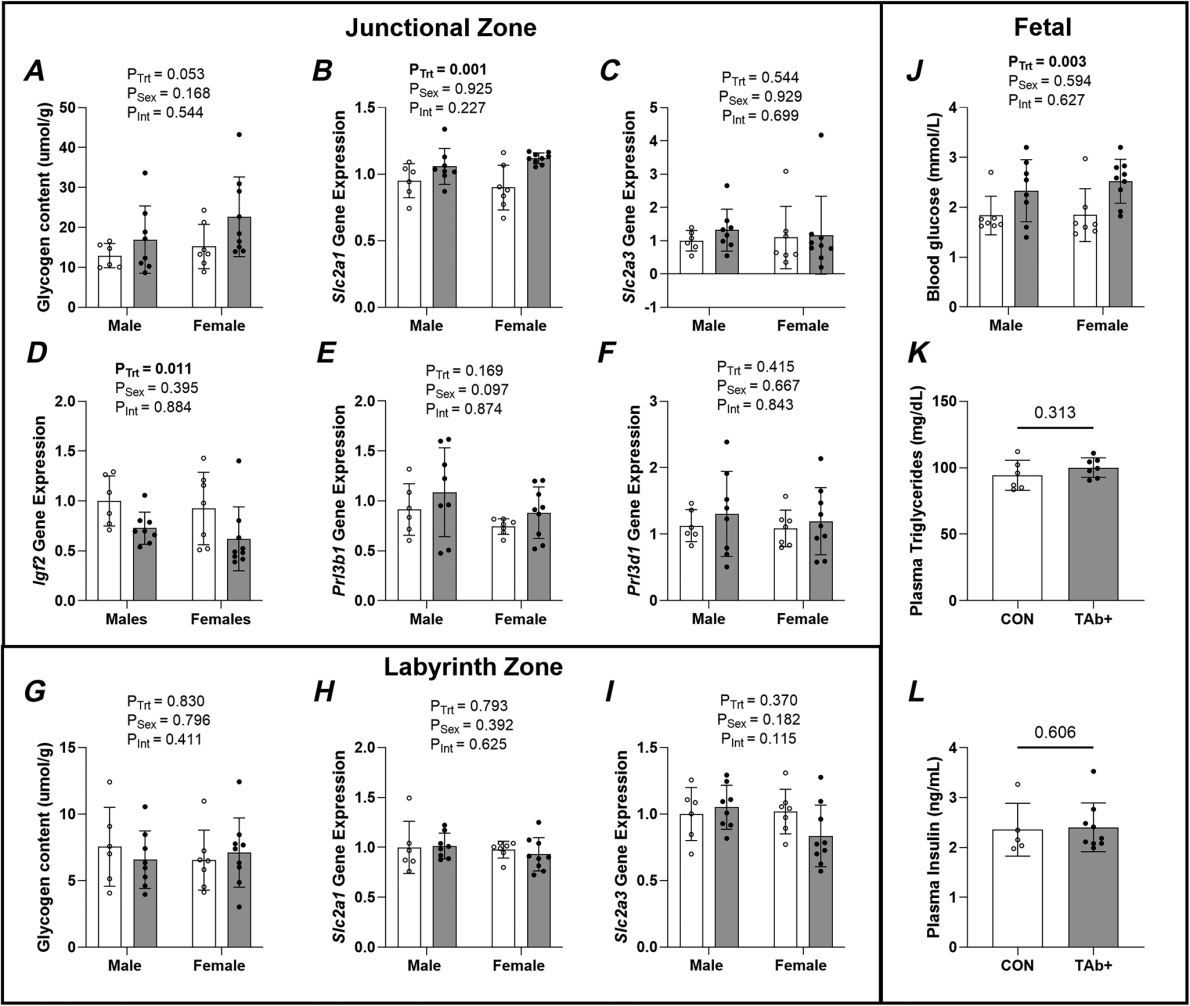
The effect of maternal TAb+ on placental and fetal metabolism Placental glycogen content (µmol/g) was assessed in junctional zone (JZ) (*A*) and labyrinth zone (LAB) (*G*) at embryonic day 20. Relative expression of key genes related to metabolism were assessed in both JZ (*B*)–(*F*) and LAB (*H*) and (*I*), including glucose transporter 1 (Slc2a1) (*B*) and (*H*), glucose transporter 3 (Slc2a3) (*C*) and (*I*), insulin‐like growth factor 2 (Igf2) (*D*), prolactin‐3b1 (Prl3b1) (*E*) and prolactin‐3d1 (Prl3d1) (*F*). All gene expression is normalised to the geomean of Actb and Tbp1. Fetal samples were measured for random blood glucose levels (mmol/L) (*J*), plasma triglycerides (mg/dL) (*K*) and plasma insulin (ng/mL) (*L*). For (*K*) and (*L*) fetal plasma samples from the same litter were pooled due to limited sample volumes (CON *n* = 5–6, TAb+ *n* = 7–9). All data points in (*A*)–(*J*) are representative male and female samples from each litter. Pups and placentas from control dams indicated by white bars, *n* = 6–10/sex. Pups and placentas from TAb+ dams indicated by grey bars, *n* = 7–10/sex. All data are presented as scatter plots with means ± SD represented. Data in (*A*)–(*J*) analysed using two‐way ANOVA with treatment and sex as factors and Sidak's multiple comparisons test (pairwise within sex). Data in (*K*) and (*L*) were compared using unpaired *t* tests with Welch's correction if data were normally distributed. Non‐normally distributed data were analysed using Mann–Whitney U tests. *P* < 0.05 was considered statistically significant.

As maternal TAb+ led to increased placental JZ weight and area, a variety of markers related to glucose metabolism and storage were assessed in the placental JZ. While glycogen content within the JZ was not affected by TAb+ (P_Trt_ = 0.053, Fig. 8*A*), several key genes related to glucose transport and storage were still assessed given the increase in JZ weight. TAb+ increased mRNA expression of the glucose transporter *Slc2a1* (P_Trt_ = 0.001, Fig. 8*B*) but not *Slc2a3* in the JZ (P_Trt_ = 0.544, Fig. 8*C*). TAb+ was also associated with increased expression of *Igf2*, a marker of growth and metabolism (P_Trt_ = 0.011, Fig. 8*D*). As TAb+ increased circulating placental lactogen, we measured mRNA expression of seven of the 25 rat prolactin‐like genes in the JZ (Alam et al., 2006; Simmons et al., 2008). TAb+ did not impact expression of genes analogous to human placental lactogen (*Prl3b1* and *Prl3d1* shown in Fig. 8*E*,*F*) or any of the other PRL genes measured (*Prl3d4*, *Prl8a4*, *Prl8a5*, *Prl8a7*, *Prl8a9*). No differences were seen in glycogen content of placental LAB (P_Trt_ = 0.830, Fig. 8*G*), and *Slc2a1* and *Slc2a3* were not affected by treatment in placental LAB (Fig. 8*H*,*I*). Fetal blood glucose values were higher in the TAb+ group compared with controls (P_Trt_ = 0.003, Fig. 8*J*). Fetal plasma TGs (*P* = 0.313, Fig. 8*K*) and insulin (P^M^ = 0.606, Fig. 8*L*) were not affected by treatment. No other markers of glucose metabolism were explored in the fetus.

### The effect of maternal TAb+ on markers of vascularisation and inflammation in placental labyrinth

**Figure 9:**
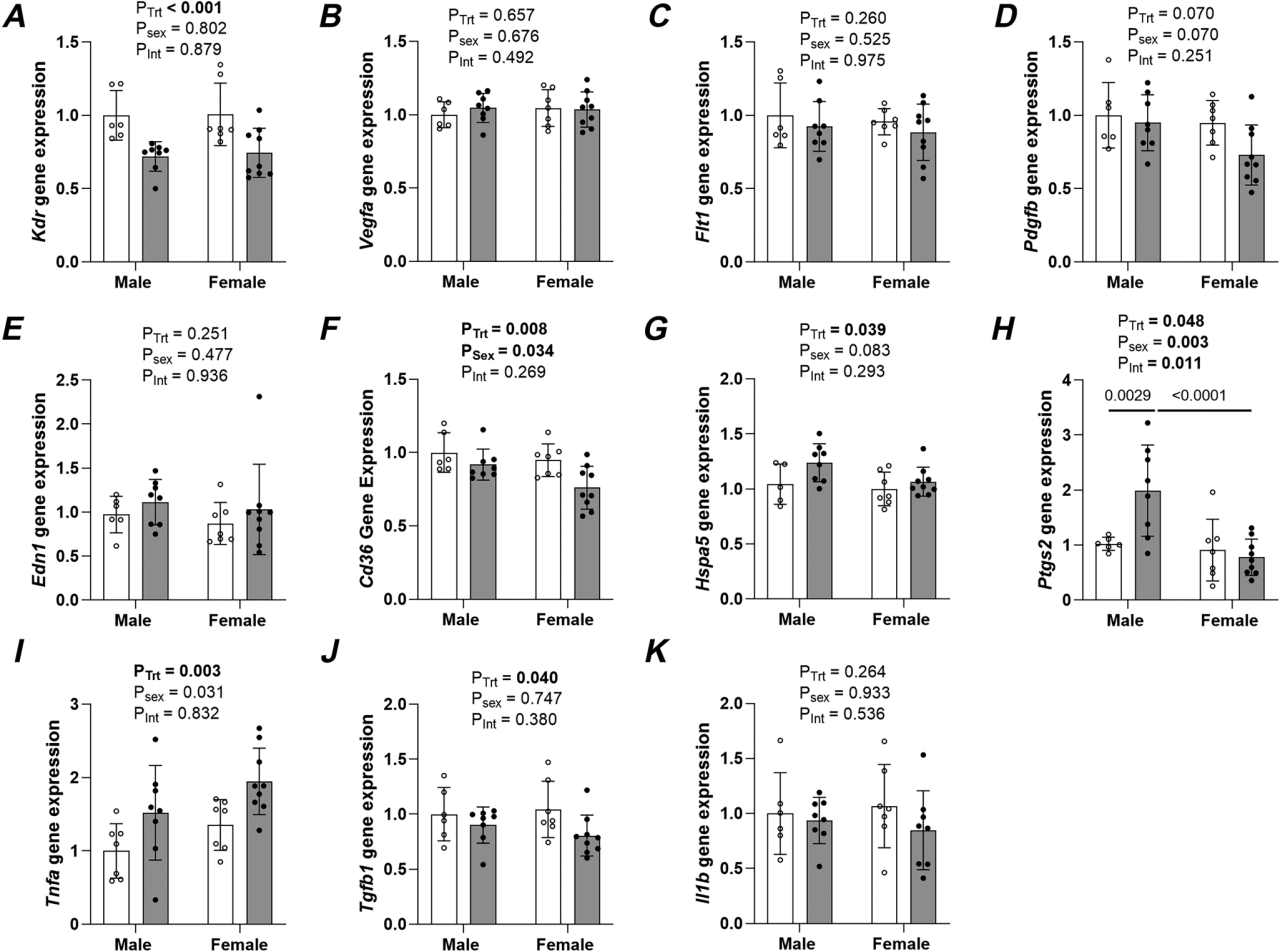
The effect of maternal TAb+ on placental expression of inflammation and vascularisation genes Relative expression in placental labyrinth of key genes involved in vascularisation and inflammation are presented in (*A*)–(*K*), including vascular endothelial growth factor receptor 2 (Kdr) (*A*), vascular endothelial growth factor A (Vegfa) (*B*), soluble fms related receptor tyrosine kinase 1 (Flt1) (*C*), platelet‐derived growth factor b (Pdgfb) (*D*) endothelin‐1 (Edn1) (*E*), platelet glycoprotein 4 (Cd36) (*F*), heat shock protein family A (Hspa5) (*G*), prostaglandin synthase 2 (Ptgs2) (*H*), tumour necrosis factor α (Tnfa) (*I*), transforming growth factor b1 (Tgfb1) (*J*) and interleukin‐1‐b (Il1b) (*K*). All gene expression is normalised to the geomean of Actb and Tbp1 or B2M and Rpl19. All data points are representative male and female samples from each litter. Pups from control dams indicated by white bars, *n* = 6–7/sex. Pups from TAb+ dams indicated by grey bars, *n* = 6–9/sex. All data are presented as scatter plots with means ± SD represented. Data analysed using two‐way ANOVA with treatment and sex as factors and Sidak's multiple comparisons test (pairwise within sex). *P* < 0.05 was considered statistically significant.

Given that TAb+ dams had reduced placental LAB relative to total area, a variety of markers related to placental vascularisation and inflammation were explored. The only gene related to vascularisation that was altered by maternal TAb+ was *Kdr*, with placentas exposed to maternal TAb+ exhibiting decreased expression (P_Trt_ < 0.001, Fig. 9*A*). *Vegfa* (P_Trt_ = 0.657, Fig. 9*B*), *Flt1* (P_Trt_ = 0.260, Fig. 9*C*) and *Pdgfb* (P_Trt_ = 0.070, Fig. 9*D*) mRNA expression was not affected by TAb+. Given the increase in plasma ET‐1 in TAb+ dams, *Edn1* mRNA expression was measured within placental LAB, with no effect of TAb+ seen (P_Trt_ = 0.251, Fig. 9*E*).

Finally, as placental inflammation is foundational to many pregnancy complications, markers of inflammation and stress were evaluated within placental LAB. While CD36 was initially assessed to examine lipid transporter mRNA expression, it also has important secondary roles in immune cell regulation and angiogenesis. Maternal TAb+ decreased placental LAB expression of *Cd36* (P_Trt_ = 0.008, Fig. 9*F*), and *Cd36* expression was also lower in female placentas compared with male placentas (P_Sex_ = 0.034, Fig. 9*F*). The expression of *Hspa5*, a key gene for endoplasmic reticulum stress and cell survival, was increased by maternal TAb+ (P_Trt_ = 0.039, Fig. 9*G*). Placental LAB mRNA expression of *Ptgs2*, a prostaglandin synthase involved in inflammation and cell proliferation, was increased by maternal TAb+ only in male placentas (P_Trt_ = 0.048, P_Int_ = 0.011, Fig. 9*H*). Finally, TAb+ dams also had increased mRNA expression of *Tnfa* (P_Trt_ = 0.003, Fig. 9*I*) and decreased expression of *Tgfb1* (P_Trt_ = 0.040, Fig. 9*J*) but no changes to *Il1b* (P_Trt_ = 0.264, Fig. 9*K*).

## Discussion

Multiple clinical studies have demonstrated a link between TAb+ and pregnancy complications such as GDM and miscarriage, but mechanistic literature is lacking. This study was designed to explore potential mechanisms underlying these relationships using pregnant rats with TAb+ but no change to TSH or T4. We demonstrate that TAb+ had minimal impacts on maternal glucose homeostasis during pregnancy, although fetuses from TAb+ dams did have elevated blood glucose concentrations at E20. TAb+ rats had significantly longer oestrous cycles before pregnancy. This coupled with increased rPL at two different timepoints and reduced progesterone at E20 suggest impairments in the regulation of key hormones required for optimal fecundity and fertility. The most striking effects associated with TAb+ in this study were on the placenta, with a larger cross‐sectional area seen in placentas exposed to TAb+. This difference was primarily seen in the JZ, both in terms of its absolute area and as a percentage of total placental area. Furthermore, key markers of glucose storage and transport were altered within JZs from TAb+ placentas. Fetal weights and litter size were not different between groups, although TAb+ dams had fewer male fetuses per litter. Finally, several important genes related to inflammation and vascular function were altered in placental LAB. This study demonstrates that although TAb+ is not associated with overt changes to maternal glucose control in our model, impaired oestrous cycling and placental development in TAb+ rats occurred despite no changes to thyroid hormone concentrations. This highlights the importance of acknowledging TAbs as an independent risk factor and focus point for treatment in addition to restoring thyroid hormones in patients planning a pregnancy.

This study utilised a rat model of TAb+, induced by immunisation with porcine thyroglobulin and FA. Using this method, we generated rats that had elevated TgAbs and TPOAbs at the time of tissue collection in late pregnancy. As TgAbs were also elevated prior to mating, continuous exposure to TAbs was likely throughout pregnancy. Thyroid morphology was assessed by an expert veterinary pathologist, and combination with TUNEL staining for apoptosis confirmed no thyroid damage had occurred by the time of tissue collection at E20. We also found no change in TSH concentrations. There were contradicting results regarding the T4 status of TAb+ animals at late pregnancy, with ELISA measurements suggesting a ninefold increase in T4, but LC–MS/MS indicated that T4 concentrations were unchanged. The method used in our study to induce TAb+ has been reported to result in varying thyroid phenotypes depending on the strain of rodent used and the duration of TAb+ before phenotype assessment. These studies mostly use non‐pregnant animals and show that the longer the TAb+ exposure, the more likely an impact on thyroid physiology (Hou et al. 2018; Song et al., 2011). As such, the key difference between our study and those undertaken previously is that we investigated outcomes at a timepoint were TAb+ has occurred, but prior to any overt thyroid dysfunction, and we studied pregnant animals. It is important to note that there are several well‐documented limitations to using ELISA‐based methods for measuring thyroid hormones with it generally acknowledged that LC–MS/MS‐based methods are more specific and accurate (Singh & Kaur, 2016). One major concern is the fact that a range of endogenous TAbs compete with the antibodies used in the ELISA and thus significantly affect the results of the assay (D'Aurizio et al., 2023). Given that we intentionally created antibodies in plasma that likely interfere with the validity of the assay, we repeated the T4 analysis using a LC–MS/MS. The LC–MS/MS results are therefore more likely to reflect the biology of the pregnant rats used in this study.

### The effect of TAb+ on maternal glucose tolerance

In women with subclinical hypothyroidism, TAb+ may increase the risk of developing GDM at lower TSH thresholds (Kent et al., 2021). We demonstrate in the current study that TAb+ is associated with subtle changes in maternal glucose control that did not lead to a GDM‐like phenotype. Patients with TAb+ and GDM may have other underlying risk factors that contribute to these outcomes. As such, it is possible that a ‘second hit’, such as pre‐existing obesity or a poor diet in the pre‐ and perinatal period, is required to unmask the effects of TAbs on glucose control in pregnancy. One related key finding was that TAb+ rats had altered concentrations of hormones known to regulate maternal glucose homeostasis in pregnancy, including an increase in rPL and a decrease in progesterone. Although we are the first to demonstrate this relationship, changes to these hormones are often linked with elevated glucose (Li et al., 2020), reduced fertility and poorer pregnancy outcomes (Bataa et al., 2024; Sibiak et al., 2020). Therefore, we propose that any clinical relationship between TAb+ and poor glucose control that is independent of T4 or TSH may relate to changes to other metabolic hormones. It is quite possible that the relationship between TAbs and metabolic health may be more representative of a complex endocrine–reproductive environment such as in women with polycystic ovary syndrome (PCOS), rather than a strictly glucose‐related phenotype.

We previously reported a GDM‐like phenotype in a methimazole‐induced model of overt hypothyroidism, mediated by reduced JZ gene expression of *Prl*‐like peptides and maternal plasma rPL. This culminated in reduced β‐cell expansion, lower insulin concentrations and glucose intolerance during an ipGTT (Kent et al., 2022). In pregnancy, placental lactogen reduces insulin sensitivity (Anthony et al., 1998; Handwerger & Freemark, 2000), increases lipolysis (Handwerger & Freemark, 2000), stimulates pancreatic islet growth and increases insulin secretion (Brelje et al., 1993). In contrast to our previous paper, we found that TAb+ was associated with increased rPL at E16 and E20 but not changes in expression of the *Prl*‐like peptide genes. As the increased rPL was maintained even after normalising to placental weight, it is unlikely that the larger placental weights in our TAb+ group were driving the changes to rPL. While the genes explored within this study were not altered by treatment, it is possible that changes to the expression of other *Prl*‐like peptide genes may be driving this increase in maternal plasma rPL concentrations, or it may be a consequence of altered clearance. The seven most important rPL genes were examined in our study, with no differences observed; however, we cannot rule out changes in the other related genes. This is a limitation of our study and could be assessed more accurately with RNA sequencing techniques, but this was outside of the scope of our study.

### The effect of TAb+ on maternal reproductive, hepatic and vascular biology

TAb+ is known to be more prevalent in women with impaired reproductive function. Both TPOAbs and TgAbs are detectable in ovarian follicular fluid and higher concentrations of TAbs are linked to changes in key metabolites in ovarian fluid (Bastos et al., 2023). Medenica et al. (2018) demonstrated that even in the absence of changes to T4 and TSH, both TgAbs and TPOAbs were elevated in the follicular fluid of women undergoing assisted reproductive technology, which was associated with a halving of pregnancies initiated per cycle and per embryo transfer. Women with PCOS exhibit higher serum TPOAb concentrations than women without PCOS (Rasoulizadeh et al., 2025). Furthermore, in women with PCOS, TPOAb concentrations correlate with concentrations of various reproductive hormones (Sharma et al., 2022). In the current study, TAb+ rats had increased oestrous cycle length and decreased plasma progesterone at E20. In rodent pregnancies, progesterone is produced from the ovary rather than the placenta, so the reduction in progesterone seen in TAb+ rats is likely an ovarian phenotype that existed before pregnancy. Unfortunately, progesterone concentrations were not examined prior to pregnancy due to limited sample volume, and ovaries were not collected, so assessment of ovarian morphology was not possible. As such, it cannot be determined whether the impact on oestrous cycle was related to changes in hormones prior to pregnancy. Future studies should focus on determining the mechanisms by which TAbs interfere with oestrous cycle length and the consequences for ovulation and pregnancy success.

Several other important maternal systems were evaluated to determine whether maternal adaptation during pregnancy was impacted by TAb+, as this is not well characterised in the literature. In our model, there were limited effects of TAb+ on the maternal vascular system, although investigations into this system were superficial. Of note, TAb+ rats did have elevated ET‐1 concentrations at E20, a marker of endothelial dysfunction. Clinical studies show elevated plasma ET‐1 can be a predictor of hypertension in pregnancy (Lu et al., 2017), although blood pressure was not assessed in our study. Maternal liver function was also explored given its role in mediating maternal–fetal fuel supply, nutrient storage and glucose generation. TAb+ was associated with increased liver weight relative to total body weight, but there were minimal changes to the markers of liver function assessed. TAb+ was not associated with liver fibrosis in this model, but TAb+ rats did have increased expression of the glucose transporter *Slc2a1*. This increase in *Slc2a1* expression matches what was also seen in placental JZ, which may suggest subtle changes to glucose transport throughout maternal and placental tissues.

### The effect of TAb+ on fetal and placental outcomes

Live birth rates in women with TAb+ undergoing *in vitro* fertilisation or intracytoplasmic sperm injection are 27% lower compared with healthy women, while the miscarriage rate is 1.4‐fold higher (Busnelli et al., 2016). Despite the disruption to oestrous cycling in the current model, TAb+ females conceived, with no differences in resorption number or litter size seen at E20. In previous animal models, TPOAb+ dams had impaired embryo implantation and increased fetal abortion rate in early (E4.5) and mid (E14) gestation (Lee et al., 2009; Wu et al., 2019), which may be more sensitive timepoints for detecting early resorptions. It is worth noting that while there was no difference in overall litter size, the proportion of males was reduced in TAb+ litters at E20. As discussed above, TAbs can accumulate in ovarian follicular fluid, where they may interfere with oocyte development and quality. This could impair embryo quality, although whether this is a sex‐specific issue is not well understood. Male embryos develop slightly faster than females and exhibit reduced survival under conditions of embryo–uterine asynchrony, reflecting a heightened sensitivity to alterations in uterine receptivity (reviewed by Gardner et al., 2010). TgAbs are known to accumulate in the endometrium, where they are associated with reduced receptivity of the uterus and implantation failure (Wu et al., 2019). Therefore, it is possible that TAbs impact uterine receptivity and thus implantation of male embryos to a greater extent than females. Given that resorption number was not different between TAb+ and control dams, it is more likely that male fetuses were lost during the early stages of implantation. Several studies have also highlighted sex‐specific differences in fetal growth in response to maternal inflammation in pregnancy complications like GDM and pre‐eclampsia (Stark et al., 2009; Meakin et al., 2022). It is possible that TAb+ dams had increased inflammation, particularly early in gestation during implantation and embryo development, that may have led to a sex‐specific survival rate. One limitation of this study was that FA was delivered only to the TAb+ animals, so the impact of adjuvant on the immune response and inflammatory environment cannot be determined. While a saline control group is commonly used in these animal models, a key priority for future pregnancy studies should be to include an FA control. In humans, sex‐specific responses to maternal inflammatory insults have been primarily related to increased androgens in males, to promote rather than prevent growth (Meakin et al., 2022). Maternal androstenedione concentrations were not altered in our TAb+ rats, although fetal and placental androgen signalling was not examined. Given that previous rodent models of TAbs in pregnancy have not examined sex‐specific differences, it is essential that future studies include sex to determine whether this result is repeatable.

While the reduction in male fetuses from TAb+ litters is noteworthy, there were limited impacts on fetal growth overall. However, there were overt changes to placental morphology and changes in the expression of genes involved in ensuring optimal placental function. Such alterations within the placenta may have compensated for any impacts to fetal size but may have induced more subtle deficits undetectable until later in life. The reduction in *Igf2* expression within placental JZ is noteworthy given that *Igf2* has previously been shown to regulate maternal glucose availability and placental metabolism to protect fetal growth (Lopez‐Tello et al., 2023). It is possible that the reduced *Igf2* expression may have prevented changes to fetal growth in our model, although plasma IGF2 levels in maternal and fetal circulation were not examined in our study.

As the key regulator of placental transport to the fetus, it was also important to assess placental LAB, with a focus on inflammation and vascularisation within this tissue. While the relative gene expression of *Flt1* and *Vegfa* were not affected, the expression of *Kdr* and *Tgfb1* was reduced due to maternal TAb+. VEGF, PDFB and TGF‐β are known to regulate the formation, stabilisation and maturation of vascular networks (reviewed in Jain, 2003), and all play a role in the regulation of placental angiogenesis through a variety of mechanisms (Helske et al., 2001; Hoodless & Wrana, 1998; Ohlsson et al., 1999). Increased placental weight and changes to maternal blood spaces and the fetal network, indicative of insufficient nutrient exchange, have been reported with reduced *Vegfa* and *Pdgfb* expression in the LAB (Natale et al., 2018). Total blood space in placental LAB was not affected by TAbs, although the proportion of maternal to fetal blood space were not directly examined. A priority for future studies should be to include an earlier stage, around mid‐gestation, as a more sensitive timepoint for detecting changes in placental vascular development, as well as examining maternal and fetal blood spaces individually.

Markers of placental inflammation were also investigated in placental LAB tissue. While the relative expression of *Il1b* was not affected, the expression of *Hspa5* and *Tnfa* were both increased in placentas exposed to maternal TAbs. TNFα is an important regulator of inflammation, apoptosis, and proliferation in the placenta, but higher levels are associated with severe pregnancy complications such as miscarriage and pre‐eclampsia (Haider & Knofler, 2009). A recent rodent model studied the impact of maternal immobilisation stress on pregnancy health, demonstrating increased TNF in the placenta as a consequence of oxidative stress (Kaya et al., 2023). While oxidative stress was not directly examined in our model, *Hspa5* was significantly elevated in placentas exposed to TAb+. As *Hspa5* is a marker of endoplasmic reticulum stress (reviewed in Lee, 2005), this may suggest a potential oxidative stress link, given the relationship between increased endoplasmic reticulum stress and oxidative stress (Gao et al., 2012). Finally, two other important placental LAB genes that were impacted by maternal TAb+ were *Cd36* and *Ptgs2*, both involved in the metabolism of arachidonic acid to prostaglandins (Chen et al., 2024). The PTGS2 result is noteworthy, given that maternal TAb+ increased placental *Ptgs2* expression only in male placentas. PTGS2, also known as cyclooxygenase 2, is increased in thyroids from patients with Hashimoto's thyroiditis, although the relationship is not well characterised (Cornetta et al., 2009). Increased *Ptgs2* expression has previously been linked with reproductive complications (Sales & Jabbour, 2003), and previous animal models of pre‐eclampsia show PTGS2 mediates the inflammatory response (Li et al., 2022). As *Ptgs2* expression was only increased in male placentas exposed to maternal TAbs, there may be a connection to the reduced male survival seen in TAb+ litters, but this needs to be explored in more detail.

### Conclusion

This study demonstrated that TAb+ in rats is associated with disrupted oestrous cycling, altered circulating reproductive and placental hormones in pregnancy, a shift in fetal sex ratios and impacted placental development. In this model, TAb+ did not lead to a dysfunctional glucose phenotype as seen in other diabetic models. Together, these findings support the concept that TAb+ contributes to altered endocrine and placental metabolic adaptations, which may have consequences for fetal development and long‐term health outcomes. Future research focusing on the isolated effects of TAbs on reproductive and metabolic function is crucial to understand how all aspects of thyroid autoimmunity may impair maternal and fetal health, and to create more targeted and effective treatments for pregnant women with high TAbs.

## Additional information

## Competing interests

None declared.

## Author contributions

R.L.B., N.L.K. and J.S.M.C. were responsible for the conception and design of the experiments. R.L.B., N.L.K., A.R., L.P., A.S.M., D.A.Z., D.M., E.S.N., M.D.W., H.B.O., J.L.M. and J.S.M.C. were each involved in data acquisition. R.L.B., N.L.K., A.R., H.B.O. and J.S.M.C. were involved in analysis and interpretation of the data. R.L.B., N.L.K. and J.S.M.C. drafted the article. All authors contributed to and approved the final version of the manuscript.

## Funding

Funding for this project was from the American Thyroid Association (Project ID: 2021‐0000000211). J.L.M. was funded by an ARC Future Fellowship (Level 3; FT170100431) and an NHMRC Investigator Grant (Leader 2; GNT2041967). A.S.M. was funded by a National Heart Foundation Postdoctoral Fellowship (108157‐2024_PDF). This research was supported by the Commonwealth through Australian Government Research Training Program Scholarships (https://doi.org/10.82133/C42F‐K220) awarded to R.L.B., N.L.K., D.A.Z. and E.S.N.

## Generative AI statement

No generative artificial intelligence tools were used in the preparation of this manuscript.

## Supporting information


Peer Review History

## Data Availability

All data supporting the results are available from the authors upon reasonable request.
