# Supplementary material for: Thyroid antibody positivity in pregnancy is associated with altered reproductive hormone concentrations and placental growth in Lewis rats
Source: J Physiol. 2026 Jul 25;604(17):7464–89. doi: 10.1113/JP291573 (PMC13532974; doi:10.1113/JP291573)
Supplement: Supplementary file 1 [file TJP-604-7464-s001.pdf]

The Journal of Physiology

Thyroid antibody positivity in pregnancy is associated with altered reproductive hormone concentrations and placental growth in Lewis rats

Rebecca L Brady, Nykola L Kent, Anna G Reid, Liana P Paszkowski, Ashley Meakin, Dayna A Zimmerman, Dinithi T Mahaliyanage, Elliott S Neal, Michael D Wiese, Helle Bielefeldt-Ohmann, Janna L Morrison, and James S M Cuffe
DOI: 10.1113/JP291573

Corresponding author(s): James Cuffe (j.cuffe1@uq.edu.au)

The following individual(s) involved in review of this submission have agreed to reveal their identity: Owen R Vaughan (Referee #1); Sarah Marshall (Referee #2)

Review Timeline:

Submission Date:	13-Apr-2026
Editorial Decision:	12-May-2026
Revision Received:	31-May-2026
Accepted:	16-Jun-2026

Senior Editor: Laura Bennet

Reviewing Editor: Christopher Lear

Transaction Report:

(Note: With the exception of the correction of typographical or spelling errors that could be a source of ambiguity, letters and reports are not edited. Depending on transfer agreements, referee reports obtained elsewhere may or may not be included in this compilation. Referee reports are anonymous unless the Referee chooses to sign their reports.)

Dear Dr Cuffe,

Re: JP-RP-2026-291573 "Thyroid antibody positivity in pregnancy is associated with altered reproductive hormone concentrations and placental growth in Lewis rats" by Rebecca L Brady, Nykola L Kent, Anna Grace Reid, Liana Paige Paszkowski, Ashley Meakin, Dayna A Zimmerman, Dinithi T Mahaliyanage, Elliott S Neal, Michael D Wiese, Helle Bielefeldt-Ohmann, Janna L Morrison, and James S M Cuffe

Thank you for submitting your manuscript to The Journal of Physiology. It has been assessed by a Reviewing Editor and by 2 expert referees and we are pleased to tell you that it is acceptable for publication following satisfactory revision.

The referee reports are copied at the end of this email.

Please address all the points raised and incorporate all requested revisions or explain in your Response to Referees why a change has not been made. We hope you will find the comments helpful and that you will be able to return your revised manuscript within 4 weeks. If you require longer than this, please contact journal staff: jp@physoc.org.

Your revised manuscript should be submitted online using the link in your Author Tasks Link Not Available. This link is accessible via your account as Corresponding Author; it is not available to your co-authors. If this presents a problem, please contact journal staff (jp@physoc.org). Image files from the previous version are retained on the system. Please ensure you replace or remove any files that are being revised.

If you do not wish to submit a revised version of your manuscript, you must inform our journal staff (jp@physoc.org) or reply to this email to request withdrawal. Please note that a manuscript must be formally withdrawn from the peer review process at one journal before it may be submitted to another journal.

TRANSPARENT PEER REVIEW POLICY: To improve the transparency of its peer review process The Journal of Physiology publishes online, as supporting information, the peer review history of all articles accepted for publication. Readers will have access to decision letters, including Editors' comments and referee reports, for each version of the manuscript, as well as any author responses to peer review comments. Referees can decide whether or not they wish to be named on the peer review history document.

ABSTRACT FIGURES: Authors are expected to use The Journal's premium BioRender account to create/redraw their Abstract Figures. Information on how to access this account is here: https://physoc.onlinelibrary.wiley.com/journal/14697793/biorender-access.

This will enable Authors to create and download high-resolution figures. If authors have used the free BioRender service, they can use the instructions provided in the link above to download a high-resolution version suitable for publication.

The link provided should only be used for the purposes of this submission. Authors will be charged for figures created on this account if they are not related to this manuscript submission.

LANGUAGE EDITING AND SUPPORT FOR PUBLICATION: If you would like help with English language editing, or other article preparation support, Wiley Editing Services offers expert help, including English Language Editing, as well as translation, manuscript formatting, and figure formatting at www.wileyauthors.com/eeo/preparation. You can also find resources for Preparing Your Article for general guidance about writing and preparing your manuscript at www.wileyauthors.com/eeo/prepresources.

RIGOUR AND REPRODUCIBILITY: We recommend authors consult The Journal's Rigour and Reproducibility requirements to ensure that their manuscript is suitable for publication: https://physoc.onlinelibrary.wiley.com/pb-assets/hub-assets/physoc/documents/TJP-Rigour-and-Reproducibility-Requirements.pdf

REVISION CHECKLIST:

Check that your Methods section conforms to journal policy: https://jp.msubmit.net/cgi-bin/main.plex?form_type=display_requirements#methods

Check that data presented conforms to the statistics policy: https://jp.msubmit.net/cgi-bin/main.plex?form_type=display_requirements#statistics

Upload a full Response to Referees file. To create your 'Response to Referees' copy all the reports, including any comments from the Senior and Reviewing Editors, into a Microsoft Word, or similar, file and respond to each point, using font or background colour to distinguish comments and responses and upload as the required file type.

Please upload two versions of your manuscript text: one with all relevant changes highlighted and one clean version with no changes tracked. The manuscript file should include all tables and figure legends, but each figure/graph should be uploaded as separate, high-resolution files. The journal is now integrated with Wiley's Image Checking service. For further details,

see: https://www.wiley.com/en-us/network/publishing/research-publishing/trending-stories/upholding-image-integrity-wileys-image-screening-service

You may also upload:

- 'Potential Cover Art' for consideration as the issue's cover image
- Appropriate Supporting Information (Video, audio or data set: see https://jp.msubmit.net/cgi-bin/main.plex?form_type=display_requirements#supp).

We look forward to receiving your revised submission.

If you have any queries, please reply to this email and we will be pleased to advise.

Yours sincerely,

Laura Bennet
Senior Editor
The Journal of Physiology

REQUIRED ITEMS

- Author photo and profile. First or joint first authors are asked to provide a short biography (no more than 100 words for one author or 150 words in total for joint first authors) and a portrait photograph. These should be uploaded and clearly labelled together in a Word document with the revised version of the manuscript. See Information for Authors for further details.

- Your manuscript must include a complete Additional Information section, including competing interests; funding; author contributions and acknowledgements.

- The Journal of Physiology funds authors of provisionally accepted papers to use the premium BioRender site to create high resolution schematic figures. Follow this link and enter your details and the manuscript number to create and download figures. Upload these as the figure files for your revised submission. If you choose not to take up this offer, we require figures to be of similar quality and resolution. If you are opting out of this service to authors, state this in the Comments section on the Detailed Information page of the submission form. The link provided should only be used for the purposes of this submission. Authors will be charged for figures created on this premium BioRender account if they are not related to this manuscript submission.

- Please upload separate high-quality figure files via the submission form.

- Please ensure that any tables are editable and in Word format, and wherever possible, embedded in the article file itself.

- Papers must comply with the Statistics Policy: https://jp.msubmit.net/cgi-bin/main.plex?form_type=display_requirements#statistics.

In summary:

- If n {less than or equal to} 30, all data points must be plotted in the figure in a way that reveals their range and distribution. A bar graph with data points overlaid, a box and whisker plot or a violin plot (preferably with data points included) are acceptable formats.

- If n > 30, then the entire raw dataset must be made available either as supporting information, or hosted on a not-for-profit repository, e.g. FigShare, with access details provided in the manuscript.

- 'n' clearly defined (e.g. x cells from y slices in z animals) in the Methods. Authors should be mindful of pseudoreplication.

- All relevant 'n' values must be clearly stated in the main text, figures and tables.

- The most appropriate summary statistic (e.g. mean or median and standard deviation) must be used. Standard Error of the Mean (SEM) alone is not permitted.

- Exact p values must be stated. Authors must not use 'greater than' or 'less than'. Exact p values must be stated to three significant figures even when 'no statistical significance' is claimed.

- Please include an Abstract Figure file and an Abstract Figure legend. An appropriate figure legend, which should not exceed 150 words in length, should be included in the main manuscript file. The Abstract Figure is a piece of artwork designed to give readers an immediate understanding of the research and should summarise the main conclusions. If possible, the image should be easily 'readable' from left to right or top to bottom. It should show the physiological relevance of the manuscript so readers can assess the importance and content of its findings. Abstract Figures should not merely recapitulate other figures in the manuscript. Please try to keep the diagram as simple as possible and without superfluous information that may distract from the main conclusion(s). Abstract Figures must be provided by authors no later than the revised manuscript stage and should be uploaded as a separate file during online submission labelled as File Type 'Abstract Figure'. Please also ensure that you include the figure legend in the main article file. All Abstract Figures should be created using BioRender. Authors should use The Journal's premium BioRender account to export high-resolution images. Details on how to use and access the premium account are included as part of this email.

EDITOR COMMENTS

Please also see 'Required Items' above.

Reviewing Editor:

Comments to the Author (Required):
Thank you for submitting your manuscript to The Journal of Physiology. Our reviewers have been positive about the importance of this study, and commended the authors on a well conducted study. Please address their suggestions for improvement.

REFEREE COMMENTS

Referee #1:

This study by Brady et al determines the effect of thyroid antibody positivity on maternal and fetal outcomes in pregnant rats. The aim was to identify mechanisms linking thyroid disease with pregnancy complications. The main findings are that thyroid antibody positivity increases estrous cycle length, alters maternal metabolic hormones and gene expression, alters placental morphology and alters fetal sex ratios. There were limited effects on maternal glucose metabolism or fetal growth. Nonetheless, this is an important study, which is novel to my knowledge, with implications for human health and the premise is clearly stated. The study is comprehensively performed, the methods are robust and reported in appropriate detail. Animal studies are appropriately described and ethically approved. The findings are discussed in light of existing research and limitations identified.

My specific comments are minor suggestions:

1. Consider explicitly stating in the key point summary, for non-expert readers, that thyroid antibody positivity and thyroid hormone deficiency are distinct phenomena. This is key to understanding the mechanistic importance of this research.

2. In the methods section, please describe how "several" placentas were selected per litter. Is this a random sample?

3. Considering the modest reduction in 2hr maternal insulin at GTT and increase in fetal blood glucose, did the authors consider examining the maternal pancreas? Is it feasible that thyroid antibodies or generalised increase in immunoglobulin levels cause auto-immune type damage to the pancreas and reduced insulin secretion? Could they measure C-reactive protein? Could these signs be indicative of mild "subclinical" glucose dysmetabolism in the rats, despite the normal GTT?

4. Please present estrous cycle data as a histogram with points to allow the reader to see the distribution - since this is an important effect of the thyroid antibody positivity.

5. Please provide further details in the methods on how placental zone areas were determined. Was this based on drawing

around the zone or point counting, which is more robust? I suggest focussing the results on the % areas of the Lz/Jz, rather than the absolute areas. The absolute area is dependent on how accurately the placenta was cut in half.

Referee #2:

This manuscript used a Lewis rat model to investigate how thyroid antibody positivity before pregnancy affects pregnancy physiology independent of thyroid hormone disruption. Although TAb+ did not alter maternal glucose tolerance or fetal growth, it was associated with longer estrous cycles, altered reproductive hormones, larger placentas, increased placental glucose transporter and inflammatory marker expression, and a reduced proportion of male fetuses. Overall, the findings suggest that TAb+ may affect pregnancy through changes in the placenta rather than maternal metabolic dysfunction.

This is a useful study addressing an underexplored question - how thyroid antibody positivity may affect pregnancy outcomes independent of overt thyroid hormone disruption. The work has clear potential impact because TAb+ is common in reproductive-aged women and is associated with adverse pregnancy outcomes, yet the mechanisms remain poorly understood.

The findings suggest that TAb+ may affect pregnancy through altered reproductive endocrine signalling, placental growth, glucose transport pathways and inflammatory activation, rather than through maternal glucose intolerance. The reduced proportion of male fetuses is also interesting and may point to sex-specific vulnerability, although this requires further research in the future.

The research is original and the design is strong, with TAb+ induced before pregnancy, assessment of estrous cycling, metabolic testing during pregnancy, and tissue collection in late gestation.

Abstract:

- I would suggest rewording the final sentence to be more cautious. I suggest something along the lines of 'These findings support further investigation of TAb+ as a thyroid hormone-independent contributor to pregnancy complications, with placental function emerging as a potential mechanistic target.'

Introduction is written well and sets the scene for justifying the research. I recommend the last paragraph be trimmed down to the essential information.

Methods are detailed extremely well, meeting all journal requirements for the use of animals in experiments.

Results:

- Figure 1 is blurry but I expect that is an issue with the submission portal rather than the figure itself.

Discussion is extremely thorough and cautious. I have no major issues with it. However, to improve readability, some sections could be streamlined to reduce repetition and sharpen the key take-home messages.

Conclusion:

- The conclusions are supported, particularly that TAb+ affects placental biology despite limited changes in the maternal parameters assessed.

END OF COMMENTS

Dear Professor Bennet,

Thank you and the reviewers for your valuable time in reviewing our manuscript, we appreciate your feedback and have made a number of changes to properly address the key points. We feel that this has improved the paper. Of note, we have trimmed the relevant sections that were suggested to improve readability but have also included a new figure which presents data that was originally within tables into a figure, as this was a key outcome for our study and was highlighted by reviewer #1 to be an important change. Please see response to specific comments below.

Editors

Thank you for taking the time to review our study, and for your commendations.

1. **Required items**

All required items have been checked within the manuscript, and an abstract figure with a legend and first author profiles with photos have now been provided. We have double checked and confirmed that our paper complies with the statistics policy, and the additional information section has also been updated.

Referee #1

Thank you for your time in reviewing our study, we appreciate your comments and supportive feedback.

1. **Consider explicitly stating in the key point summary, for non-expert readers, that thyroid antibody positivity and thyroid hormone deficiency are distinct phenomena. This is key to understanding the mechanistic importance of this research.**

We have altered the key points summary to better reflect this and appreciate your assistance helping us to highlight this important part of our research.

2. **In the methods section, please describe how "several" placentas were selected per litter. Is this a random sample?**

We have tweaked the wording to better reflect the random sample collected of these placentas (line 215).

3. **Considering the modest reduction in 2hr maternal insulin at GTT and increase in fetal blood glucose, did the authors consider examining the maternal pancreas? Is it feasible that thyroid antibodies or generalised increase in immunoglobulin levels cause auto-immune type damage to the pancreas and reduced insulin secretion? Could they measure C-reactive protein? Could these signs be indicative of mild "subclinical" glucose dysmetabolism in the rats, despite the normal GTT?**

This is a finding that we were also initially interested in, and your point around the potential for increased immunoglobulin levels leading to auto-immune damage to other organs is an important one. We collected and fixed the pancreas from our rats but decided not to follow up with histological examination due to the lack of an overt glucose phenotype within our model. We agree that our model has induced a subtle change in glucose physiology and have altered the wording slightly in the discussion to reflect this (line 569).

4. **Please present estrous cycle data as a histogram with points to allow the reader to see the distribution - since this is an important effect of the thyroid antibody positivity.**

We agree that changes to estrous cycling is an important finding from our study. This was not a phenotype we initially anticipated and as such, have only limited data related to this phenotype. As such, we initially included it only in the table as we didn't have addition data to put in an independent figure that was focused on fertility. We have now decided after your recommendation to create a fertility figure, including estrous cycle data in combination with litter size, number of resorptions, and male fetal survival (Figure 3). We have removed these results from the table and amended figure numbers within the results text. We highlight in the discussion that this is an important result and that future studies should explore this further (line 620).

5. **Please provide further details in the methods on how placental zone areas were determined. Was this based on drawing around the zone or point counting, which is more robust? I suggest focussing the results on the % areas of the Lz/Jz, rather than the absolute areas. The absolute area is dependent on how accurately the placenta was cut in half.**

Placental zone areas were determined by drawing around the zone, following the methods we have previously published (reference now included, line 327). While not as robust as point counting (which we have also previously undertaken), a higher magnification was used than we would employ when performing point counting to improve accuracy when identifying the border between cell types. We also acknowledge that the area is strongly impacted by how accurately the placenta is cut in half and we have careful protocols to ensure this is minimised. We have made sure to now focus more heavily on the ratios in our discussion (line 532). Importantly, we have retained both absolute area and the ratios given that they both provide important information. Using data for only the ratio would suggest that labyrinth size is decreased which is not accurate as absolute data suggest either no change or potentially an increase in size.

Referee #2

Thank you for your time in reviewing our study, we appreciate your comments and supportive feedback.

1. **This is a useful study addressing an underexplored question - how thyroid antibody positivity may affect pregnancy outcomes independent of overt thyroid hormone disruption. The work has clear potential impact because TAb+ is common in reproductive-aged women and is associated with adverse pregnancy outcomes, yet the mechanisms remain poorly understood. The findings suggest that TAb+ may affect pregnancy through altered reproductive endocrine signalling, placental growth, glucose transport pathways and inflammatory activation, rather than through maternal glucose intolerance. The reduced proportion of male fetuses is also interesting and may point to sex-specific vulnerability, although this requires further research in the future. The research is original and the design is strong, with TAb+ induced before pregnancy, assessment of estrous cycling, metabolic testing during pregnancy, and tissue collection in late gestation.**

We thank the reviewer for this overview and appreciate the support for our efforts to address this relationship.

2. **Abstract: I would suggest rewording the final sentence to be more cautious. I suggest something along the lines of 'These findings support further investigation of TAb+ as a thyroid hormone-independent contributor to pregnancy complications, with placental function emerging as a potential mechanistic target.'**

We have changed the final sentence of the abstract to match this suggestion (line 89).

3. **Introduction is written well and sets the scene for justifying the research. I recommend the last paragraph be trimmed down to the essential information.**

We have trimmed the final paragraph in the introduction where possible to make this section more succinct and focused on the essential information.

4. **Results: Figure 1 is blurry but I expect that is an issue with the submission portal rather than the figure itself.**

We will ensure that figures uploaded to the journal for submission meet the required resolution for final submission and apologise if this impeded interpretation at all.

5. **Discussion is extremely thorough and cautious. I have no major issues with it. However, to improve readability, some sections could be streamlined to reduce repetition and sharpen the key take-home messages.**

We do acknowledge that the discussion is lengthy and have taken measures to trim this where possible to improve the readability, while still including appropriate length of interpretation for key results.

Dear Dr Cuffe,

Re: JP-RP-2026-291573R1 "Thyroid antibody positivity in pregnancy is associated with altered reproductive hormone concentrations and placental growth in Lewis rats" by Rebecca L Brady, Nykola L Kent, Anna G Reid, Liana P Paszkowski, Ashley Meakin, Dayna A Zimmerman, Dinithi T Mahaliyanage, Elliott S Neal, Michael D Wiese, Helle Bielefeldt-Ohmann, Janna L Morrison, and James S M Cuffe

We are pleased to tell you that your paper has been accepted for publication in The Journal of Physiology.

The last Word (or similar) version of the manuscript provided will be used by the Production Editor to prepare your proof. When this is ready you will receive an email containing a link to Wiley's Online Proofing System. The proof should be thoroughly checked and corrected as promptly as possible.

Authors should note that it is too late at this point to offer corrections prior to proofreading. Major corrections at proof stage, such as changes to figures, will be referred to the Editors for approval before they can be incorporated. Only minor changes, such as to style and consistency, should be made at proof stage. Changes that need to be made after proof stage will usually require a formal correction notice.

All queries at proof stage should be sent to: TJP@wiley.com.

Following copyediting and typesetting the accepted version will be published online.

Yours sincerely,

Laura Bennet
Senior Editor
The Journal of Physiology

IMPORTANT POINTS TO NOTE FOLLOWING ACCEPTANCE OF YOUR PAPER:

• IMPORTANT NOTICE ABOUT OPEN ACCESS: To assist authors whose funding agencies mandate immediate public access to published research findings, The Journal of Physiology allows authors to pay an Open Access (OA) fee to have their papers made freely available immediately on publication.

The Corresponding Author will receive an email from Wiley with details on how to register or log in to Wiley Authors where you will be able to place an order.

• You can check if your funder or institution has a Wiley Open Access Account here:
https://authors.wiley.com/author-resources/Journal-Authors/open-access/author-compliance-tool.html

• TRANSPARENT PEER REVIEW POLICY: To improve the transparency of its peer review process, The Journal of Physiology publishes online as supporting information the peer review history of all articles accepted for publication. Readers will have access to decision letters, including Editors' comments and referee reports, for each version of the manuscript as well as any author responses to peer review comments. Referees can decide whether or not they wish to be named on the peer review history document.

• You can help your research get the attention it deserves! Check out Wiley's free Promotion Guide for best-practice recommendations for promoting your work at: www.wileyauthors.com/eeo/guide. You can learn more about Wiley Editing Services which offers professional video, design, and writing services to create shareable video abstracts, infographics, conference posters, lay summaries, and research news stories for your research at: www.wileyauthors.com/eeo/promotion.

• If you would like to receive our 'Research Roundup', a monthly newsletter highlighting the cutting-edge research published in The Physiological Society's family of journals (The Journal of Physiology, Experimental Physiology, Physiological Reports, The Journal of Nutritional Physiology and The Journal of Precision Medicine: Health and Disease), please click this link, fill in your name and email address and select 'Research Roundup':
https://www.physoc.org/journals-and-media/membernews

EDITOR COMMENTS

Reviewing Editor:

Comments to the Author (Required):
Thank you for your revisions. Congratulations on an excellent manuscript, and thank you for your contribution to The Journal.

REFEREE COMMENTS

Referee #1:

Thank you for responding to my comments.

Referee #2:

Thank you for taking the time to respond to my comments. I am satisfied with all revisions. Best wishes with the publication and future research.
